# The ghosts of propagation past: haplotype information clarifies the relative influence of stocking history and phylogeographic processes on contemporary population structure of walleye (*Sander vitreus*)

**DOI:** 10.1111/eva.13186

**Published:** 2021-01-29

**Authors:** Matthew L. Bootsma, Loren Miller, Greg G. Sass, Peter T. Euclide, Wesley A. Larson

**Affiliations:** ^1^ Wisconsin Cooperative Fishery Research Unit College of Natural Resources University of Wisconsin‐Stevens Point Stevens Point WI USA; ^2^ Minnesota Department of Natural Resources University of Minnesota St. Paul MN USA; ^3^ Office of Applied Science Wisconsin Department of Natural Resources Escanaba Lake Research Station Boulder Junction WI USA; ^4^ U.S. Geological Survey Wisconsin Cooperative Fishery Research Unit College of Natural Resources University of Wisconsin‐Stevens Point Stevens Point WI USA; ^5^ Present address: Ted Stevens Marine Research Institute Alaska Fisheries Science Center National Marine Fisheries Service National Oceanic and Atmospheric Administration Juneau AK USA

**Keywords:** co‐ancestry, microhaplotype, population structure, RAD sequencing, stocking, walleye

## Abstract

Stocking of fish is an important tool for maintaining fisheries but can also significantly alter population genetic structure and erode the portfolio of within‐species diversity that is important for promoting resilience and adaptability. Walleye (*Sander vitreus*) are a highly valued sportfish in the midwestern United States, a region characterized by postglacial recolonization from multiple lineages and an extensive history of stocking. We leveraged genomic data and recently developed analytical approaches to explore the population structure of walleye from two midwestern states, Minnesota and Wisconsin. We genotyped 954 walleye from 23 populations at ~20,000 loci using genotyping by sequencing and tested for patterns of population structure with single‐SNP and microhaplotype data. Populations from Minnesota and Wisconsin were highly differentiated from each other, with additional substructure found in each state. Population structure did not consistently adhere to drainage boundaries, as cases of high intra‐drainage and low inter‐drainage differentiation were observed. Low genetic structure was observed between populations from the upper Wisconsin and upper Chippewa river watersheds, which are found as few as 50 km apart and were likely homogenized through historical stocking. Nevertheless, we were able to differentiate these populations using microhaplotype‐based co‐ancestry analysis, providing increased resolution over previous microsatellite studies and our other single SNP‐based analyses. Although our results illustrate that walleye population structure has been influenced by past stocking practices, native ancestry still exists in most populations and walleye populations may be able to purge non‐native alleles and haplotypes in the absence of stocking. Our study is one of the first to use genomic tools to investigate the influence of stocking on population structure in a nonsalmonid fish and outlines a workflow leveraging recently developed analytical methods to improve resolution of complex population structure that will be highly applicable in many species and systems.

## 1 INTRODUCTION

Conservation of population genetic diversity and intra‐species biocomplexity has long been recognized as important to maintaining the long‐term sustainability of exploited species (Hilborn et al., 2003; Schindler et al., 2010). Exploited populations that have not been managed for genetic diversity and adaptability have collapsed more easily as the result of a temporary change in their environment, such as disease, change in forage, or increased harvest (Hutchinson, 2008). Fish species are currently experiencing unprecedented environmental conditions due to habitat loss and climate change, therefore maintaining a robust portfolio of adaptive diversity is more important than ever (Bay et al., 2018).

The benefits provided by a robust portfolio of population diversity in terms of consistent harvest and ecosystem services have been well demonstrated in a number of systems (reviewed in Schindler et al., 2015), but accurately defining the demographic and genetic variation among independent populations that comprise these portfolios is difficult (Luck et al., 2003). In natural systems, landscape connectivity, habitat heterogeneity, and differential selective pressures influence patterns of gene flow and therefore contemporary population genetic structure (hereafter referred to as population structure, Allendorf et al., 2013). However, patterns of gene flow in exploited species also may be influenced by translocation or stocking for management purposes, which can result in human‐mediated gene flow between genetically divergent populations (Laikre & Ryman, 1996; Lamaze et al., 2012; Marie et al., 2010). Depending on the context, stocking can affect natural populations in a positive (e.g., genetic rescue, increased diversity, reduction of deleterious mutations, Ferchaud et al., 2018; Whiteley et al., 2015) or negative (e.g., loss of coadapted gene complexes, Hedrick & Garcia‐Dorado, 2016; Utter, 2004) manner. Therefore, identifying genetic boundaries and understanding the relative influence of natural versus anthropogenic gene flow in systems with a history of stocking or translocations is vital for developing strategies to conserve and restore natural genetic diversity.

Exploited populations of fish represent an excellent model for investigating whether human‐mediated gene flow influences patterns of population structure, as supplemental stocking is a frequently used tool for enhancing population sizes and augmenting harvest opportunities in these species (Tingley et al., 2019). Previous studies investigating the influence of stocking on patterns of population structure in fishes have largely focused on salmonids, where classical genetic markers (primarily allozymes, mtDNA, and microsatellites) have been used to illustrate the substantial influences of human‐mediated gene flow on natural population structure (reviewed in Utter, 2004). For example, Williamson and May (2005) documented strong evidence for large‐scale genetic homogenization of fall‐run Chinook salmon (*Oncorhynchus tshawytscha*) in the Sacramento River, California, USA, using genotypes from seven microsatellite loci. As the field of population genetics has transitioned to genomics, new opportunities for testing for the influences of stocking have emerged (Allendorf et al., 2010). Genomic tools have facilitated in‐depth analysis of the consequences of human‐mediated hybridization in cutthroat and rainbow trout (*O. mykiss* and *clarkii*) (Hohenlohe et al., 2013), investigation of deleterious alleles in stocked populations of lake trout (*Salvelinus namaycush*) (Perrier et al., 2017), and analysis of fine‐scale recombination patterns to understand variable patterns of introgression across the genome in stocked populations of brook trout (*Salvelinus fontinalis*) (Leitwein et al., 2019). However, genomic tools have rarely been applied to test for the influence of stocking on genetic structure of fish species outside of salmonids (but see Rougemont et al., 2019).

Salmonids generally display higher levels of population structure than many other fish species due to high natal philopatry (Utter, 2004); this substantial population structure makes identifying human‐mediated gene flow simpler. In species with lower levels of population structure, such as marine species, species with higher population sizes, or lower natal philopatry, testing for human‐mediated gene flow is more challenging (Schall et al., 2017). Fortunately, the development of new genomic analysis tools has the potential to improve estimates of fine‐scale genetic structure in these systems (Allendorf et al., 2010). One approach that is particularly promising for delineating fine‐scale genetic structure is the use of single nucleotide polymorphism (SNP) microhaplotypes derived from genotyping‐by‐sequencing (GBS) data (reviewed in Leitwein et al., 2020). These microhaplotypes contain linkage disequilibrium information for tightly linked loci, facilitating increased resolution for investigating genetic ancestry.

We used genomic techniques, including analysis of microhaplotype data, to test for the relative influence of anthropogenic and natural processes on patterns of fine‐scale genetic structure in walleye (*Sander vitreus*) from Wisconsin and Minnesota in the upper midwestern United States. Walleye are an important cultural, economic, and recreational resource in the Midwest and are one of the most sought‐after sportfishes in the region. Recently, however, declines in natural recruitment have been observed throughout the region (Embke et al., 2019; Hansen et al., 2015; Rypel et al., 2018), which is comprised of numerous glacial lakes. These declines may be linked to increasing water temperatures (Hansen et al., 1998; Rypel et al., 2018), a trend that is anticipated to continue as a result of climate change (Pryor et al., 2014). In response to these declines, supplemental stocking programs that have existed in the region for over a century have been expanded to maintain the fishery. To support supplemental stocking, funding programs such as the Wisconsin Walleye Initiative have provided several million dollars of funding annually over the past decade. Increased stocking effort has been matched by a shift in management paradigms, expanding local management strategies that protect populations within a single lake to include regional strategies aimed at protecting and promoting ecological resilience of the fishery as a whole (Tingley et al., 2019). Given the heterogeneous yet inter‐dependent nature of fishery resources in glacial lakes, a thorough understanding of walleye genetic diversity is integral to a regionalized management approach aiming to maintain a robust portfolio of population genetic diversity for ensuring long‐term sustainability and ecological resilience.

Early studies on the genetic diversity of walleye in the region, using mtDNA, established the presence of several distinct lineages originating from multiple glacial refugia approximately 10 kya (Billington, 1996; Billington et al., 1992; Billington & Strange, 1995; Stepien et al., 2009). Additionally, differences in length‐at‐age have been observed among walleye that were from similar geographic regions but were colonized from these different glacial refugia, suggesting the potential of adaptive divergence across these lineages (Zhao et al., 2008). Despite accurate characterization of postglacial recolonization pathways having provided vital information for designing current and future studies, mtDNA markers lacked the resolution to describe the genetic boundaries among discrete stocks on the scale necessary for management to account for genetic divergence among stocks.

In an attempt to improve resolution of fine‐scale genetic structure, Hammen and Sloss (2019) analyzed genetic variation of walleye across Northern Wisconsin using a suite of ten microsatellite loci. Although Hammen and Sloss (2019) found some evidence of genetic differentiation that was consistent with historical glacial hydrology, they also observed multiple exceptions to this pattern; namely, that several populations near the Chippewa River headwaters appeared most similar to populations in the upper Wisconsin River basin. The inability to distinguish these populations with microsatellites was attributed to the effect of long‐term supplemental stocking, which has been practiced routinely in many of the lakes throughout the region for much of the 20th century. Unfortunately, much of this stocking took place with few records of the sources of stocked fish, further complicating the issue. Although no similar analysis of walleye population structure in Minnesota has been published, unpublished data (L. Miller, Minnesota Department of Natural Resources) suggested that similarly convoluted patterns of population structure exist in some regions of Minnesota as well.

Given the complexity of the distribution of genetic diversity among walleye populations throughout Wisconsin and Minnesota, our goal was to use genomic techniques to: (1) test for fine‐scale genetic boundaries across the landscape; (2) assess whether the genetic integrity of populations may have been altered due to stocking; and (3) define appropriate population genetic boundaries that can be integrated into future management plans. Our genomic analyses, especially the analysis incorporating microhaplotypes, provided increased resolution of population structure compared to previous studies based on microsatellites. This increased resolution of the intricate patterns of walleye population structure across Minnesota and Wisconsin provides information that can be used to conserve the genetic biocomplexity of walleye populations in the region.

## 2 MATERIALS AND METHODS

### 2.1 Study area and sample sites

**FIGURE 1:**
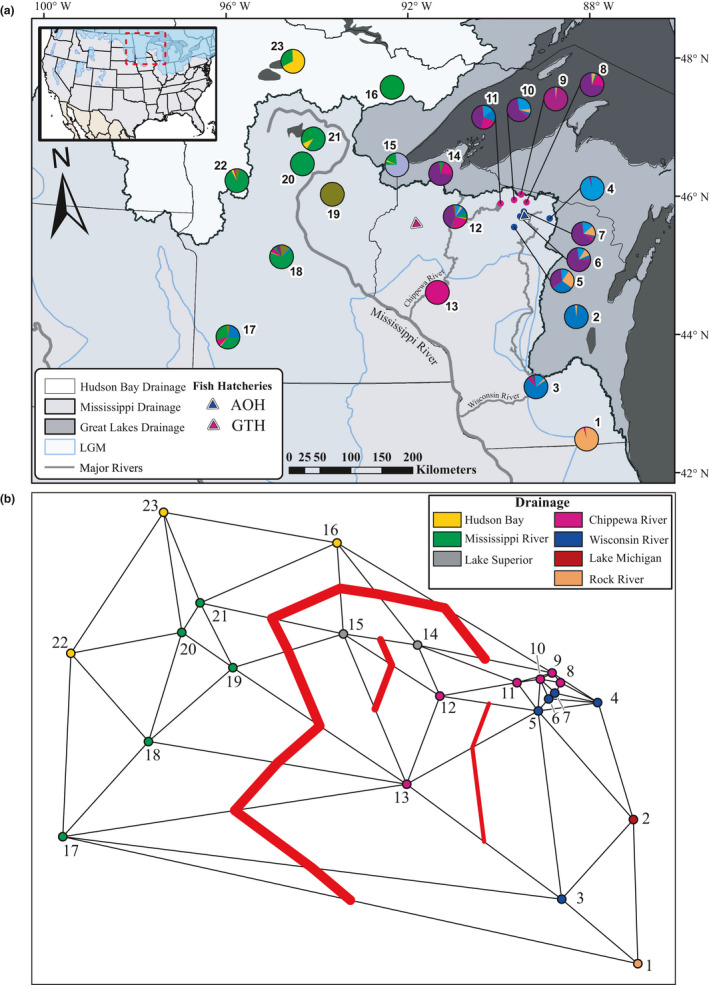
(a) Walleye (*Sander vitreus*) sample locations across Wisconsin (WI, populations #1–14), the St. Louis River (border water, #15), and Minnesota (MN, #16–23), USA. Populations are numbered according to Table 1 and represented by pie charts composed of the overall admixture proportions within the population (*K* = 11, Figure 3). Populations on the border of the upper Chippewa basin (pink dots) and upper Wisconsin basin (blue dots) are annotated with lines for clarity. Relevant hatcheries are designated with triangles (Governor Tommy Thompson, GTH; Art Oehmcke, AOH [located between #6 and #7]), and colored to denote the basin most closely associated with them. Major drainages are shown in white (Hudson Bay), light gray (Mississippi River), and dark gray (Great Lakes), and major rivers are shown with gray lines; last glacial maximum (LGM) denoted by blue outline and shading (~20 kya, see inset for full range). (b) Boundaries to geneflow among the 23 populations of walleye sampled, identified using Monmonier's algorithm. Points represent sampled populations, are spatially distributed according to their latitude and longitude, and are numbered according to Table 1. Point color represents the major drainage in which a population is found (Hudson Bay: yellow; Mississippi River, MN: green; Lake Superior: gray; Chippewa River, WI: purple; Wisconsin River, WI: blue; Rock River, WI: orange; Lake Michigan: red). Thin black lines show the connectivity network which defines neighboring populations. Red lines intersecting connections show boundaries between neighbors, with line thickness representing the order in which the boundary was identified (i.e., strength. Thickest line is strongest)

The upper midwestern United States is composed of a high density of inland, glacial lakes connected by complex dendritic habitat and intersected by three continental‐scale drainages; (1) the Hudson Bay drainage; (2) the Mississippi River drainage; and (3) the Laurentian Great Lakes drainage (see Figure 1). Large‐scale genetic variation of walleye across this landscape, as inferred from mtDNA haplotypes and microsatellites, generally reflects patterns of postglacial recolonization from three glacial refugia: the Missouri refugium, the Mississippi refugium, and the Atlantic refugium (Billington, 1996; Billington et al., 1992; Billington & Strange, 1995; Stepien et al., 2009). While egg‐take operations have been operating in the region since the late 1800s, few records of which sources were stocked into lakes were kept until about the 1990s. One well‐recorded stocking even of note, however, was the stocking of over 100 million fry from Pike River, Minnesota into Red Lake, Minnesota between 1999 and 2003 (Logsdon et al., 2016).

In the current study, we analyzed genotypes from 954 walleye sampled from 23 populations across Wisconsin and Minnesota. All samples were previously genotyped using restriction site‐associated DNA (RAD) sequencing by Bootsma et al. (2020), and represent each of the major drainages across the study range. The highest sampling effort was weighted toward the upper Chippewa and upper Wisconsin drainages in Wisconsin, two watersheds that border each other and were challenging to differentiate using microsatellites (Hammen & Sloss, 2019). Most of our samples are from lakes or from rivers during spawning season when adults from nearby lakes are present; therefore, our study system is best conceptualized as a series of largely isolated lakes rather than a dendritic and interconnected series of river populations.

### 2.2 Genomic data

DNA was extracted with Qiagen DNeasy® Blood and Tissue Kits, and RAD sequencing was conducted using the BestRAD protocol (Ali et al., 2016) and methods outlined in Bootsma et al. (2020). RAD libraries were sequenced on Illumina HiSeq 4000 machines using PE150 chemistry. Data processing to filter raw sequences, identify SNPs, and call genotypes using paired‐end reads was conducted in STACKS v2.2 (Rochette et al., 2019) with the following parameters: process_radtags (‐‐filter_illumina, ‐‐bestrad, ‐e SbfI, ‐c, ‐q, ‐r, ‐t 140), ustacks (‐‐disable‐gapped, ‐‐model_type bounded, ‐‐bound_high 0.05, ‐M 3, ‐max_locus_stacks 4, ‐m 3, ‐H), and cstacks (‐n 3). We conducted *de novo* construction of loci rather than aligning to a related reference genome as suggested by Paris et al., (2017). A raw vcf file containing genotypes for all individuals, available from Bootsma et al. (2020), was used to select SNPs for downstream analyses; this vcf was produced in the *populations* module of STACKS v2.2 with two loose filtering parameters: retain only SNPs present in >5% of individuals and a minimum minor allele frequency >0.005.

Genotypes were filtered in vcftools v0.1.15 (Danecek et al., 2011) to select high‐quality SNPs and individuals for downstream analyses. Individuals were retained only if they were genotyped at >80% of SNPs. Likewise, SNPs were retained only if they were genotyped in >80% of individuals and had a minimum minor allele count of 3. Data then were input into HDplot (Mckinney, Waples et al., 2017) to visualize allele specific read ratios and identify putative duplicate loci, removing those with *H* > 0.5 or *D* between −7 and 7. We then selected one SNP per locus, choosing the SNP with the highest minor allele frequency (MAF), selecting the first SNP when two or more SNPs at a locus had equal MAF estimates. Finally, any SNP with an *F*_IS_ estimate >0.5 was removed.

An additional dataset containing microhaplotypes was then constructed for fineRADstructure (Malinsky et al., 2018) analysis. Microhaplotypes were generated via *populations*, whitelisting only individuals and SNPs retained through filtering prior to selecting a single SNP per locus. We then used vcftools to remove any locus with >10 alleles or an estimated *F*_IS_ > 0.5. Finally, we aligned all loci to the yellow perch (*Perca flavescens*) genome (Feron et al., 2020) to improve accuracy of effective population size estimates, provide context for genome scans, and to facilitate fineRADstructure analysis, which requires alignment information. Alignments were conducted in BLASTN (Camacho et al., 2009); the best alignment for each locus was retained, and all retained alignments had *e*‐values <1e^−67^.

### 2.3 Genetic diversity and population structure

To assess genetic diversity, we estimated the observed (*H*_O_) and expected (*H*_E_) heterozygosity, allelic richness (*A*_r_), and *F*_IS_ for each sampling location using diveRsity v1.9.90 (Keenan et al., 2013). Effective population size (*N*_e_) was estimated with the bias‐corrected linkage disequilibrium method (Hill, 1981; Waples & Do, 2010; Waples, 2006) in the software package NeEstimator v2.1 (Do et al., 2014) with a *p*‐crit of 0.05 (Waples et al., 2016). To correct for physical linkage, only comparisons between loci found on different chromosomes of the yellow perch genome were included (Waples et al., 2016). Perch and walleye are both in the Percidae family and share karyotypes (Danzmann, 1979), so chromosomes should correspond between the species. *N*_e_ calculations using the linkage disequilibrium method can be biased slightly downward when individuals from multiple cohorts are included in the sample due to a slight Wahlund effect (7% downward bias on average) (Waples et al., 2014). Nonetheless, this small bias should not greatly affect the interpretation of the *N*_e_ results.

To assess patterns of genetic divergence between populations, we estimated pairwise *F*_ST_ in Arlequin v3.5.2 (Excoffier & Lischer, 2010) and tested for significance using 10,000 permutations per test (Bonferroni‐corrected α = 0.0001). We then constructed an unrooted neighbor‐joining dendrogram in poppr v2.8.2 (Kamvar et al., 2014) using Nei's Da (Nei et al., 1983) and 10,000 bootstrap replicates. Neutral genetic structure was further evaluated by performing three hierarchical AMOVAs using the following datasets: (1) all populations grouped by state, (2) Wisconsin populations grouped by major drainage, and (3) Minnesota populations grouped by major drainage (Table 1). The use of separate analyses within the states was supported by both boundary detection and clustering analyses (i.e., Monmonier's algorithm and fineRADstructure, see Results). Samples from the St. Louis River, a border water between the two states, were grouped with Wisconsin populations in AMOVA and in all further analyses where Wisconsin and Minnesota populations were analyzed separately, as this clustering was supported by both the dendrogram and fineRADstructure (see Results). AMOVAs were conducted using default parameters within Arlequin. Hierarchical groupings within each test were constructed to reflect contemporary watershed boundaries.

**TABLE 1:** Information on walleye (*Sander vitreus*) collections from 23 sites across 7 major drainages throughout Wisconsin and Minnesota, USA

Population ID	State	Major Drainage	Latitude	Longitude	*n* sampled	*n* genotyped	*N*_e_	*N*_e_ CI low	*N*_e_ CI high	*A*_r_	*H*_O_	*H*_E_	*F*_IS_	Mean intra‐population co‐ancestry score	AMOVA grouping		
															All	Wisconsin	Minnesota
(1) Delavan Lake	Wisconsin	Rock River	42.58	−88.63	48	48	154	153	155	1.584	0.165	0.167	0.016	22.662	1	1	NA
(2) Wolf River	Wisconsin	Lake Michigan	44.36	−88.69	47	41	1466	1388	1553	1.616	0.184	0.170	−0.052	22.175	1	2	NA
(3) Lake Wisconsin	Wisconsin	Wisconsin River	43.38	−89.58	48	45	1186	1148	1227	1.643	0.171	0.176	0.027	21.940	1	3	NA
(4) Medicine Lake	Wisconsin	Wisconsin River	45.81	−89.13	47	47	1471	1409	1539	1.592	0.165	0.166	0.005	22.244	1	3	NA
(5) Willow Flowage	Wisconsin	Wisconsin River	45.71	−89.87	48	48	19,781	12,866	42,739	1.628	0.167	0.173	0.040	21.442	1	3	NA
(6) Kawaguesaga Lake	Wisconsin	Wisconsin River	45.86	−89.74	48	42	3257	2968	3608	1.623	0.169	0.172	0.008	21.506	1	3	NA
(7) Big Arbor Vitae Lake	Wisconsin	Wisconsin River	45.93	−89.65	48	44	974	946	1003	1.625	0.168	0.172	0.034	21.422	1	3	NA
(8) Escanaba Lake	Wisconsin	Chippewa River	46.06	−89.59	48	44	480	473	487	1.614	0.175	0.169	−0.020	21.601	1	4	NA
(9) Sanford Lake	Wisconsin	Chippewa River	46.18	−89.69	48	44	55	55	55	1.528	0.167	0.159	−0.036	23.388	1	4	NA
(10) Manitowish Lake	Wisconsin	Chippewa River	46.11	−89.85	47	35	1372	1294	1461	1.621	0.171	0.172	0.017	21.446	1	4	NA
(11) Turtle Flambeau Flowage	Wisconsin	Chippewa River	46.06	−90.13	47	38	488	479	497	1.641	0.171	0.172	0.006	21.185	1	4	NA
(12) Chippewa Flowage	Wisconsin	Chippewa River	45.90	−91.09	47	43	889	864	916	1.633	0.173	0.171	−0.005	21.207	1	4	NA
(13) Eau Claire River	Wisconsin	Chippewa River	44.80	−91.50	47	47	937	910	964	1.568	0.159	0.160	0.008	22.897	1	4	NA
(14) Lake Millicent	Wisconsin	Lake Superior	46.53	−91.37	48	32	226	223	230	1.605	0.173	0.165	−0.032	21.627	1	5	NA
(15) St. Louis River	Border water	Lake Superior	46.65	−92.21	32	30	132	131	133	1.596	0.164	0.166	0.009	23.334	1	5	NA
(16) Pike River	Minnesota	Hudson Bay	47.59	−92.39	32	28	6772	4511	13,570	1.493	0.141	0.143	0.012	23.278	2	NA	2
(17) Sarah Lake	Minnesota	Mississippi River	44.15	−95.77	32	30	943	894	999	1.582	0.164	0.163	−0.005	22.264	2	NA	1
(18) Lake Koronis	Minnesota	Mississippi River	45.33	−94.70	32	17	94	92	95	1.566	0.155	0.153	−0.017	22.112	2	NA	1
(19) Mille Lacs	Minnesota	Mississippi River	46.25	−93.67	32	29	5671	3997	9748	1.502	0.150	0.146	−0.020	23.721	2	NA	1
(20) Pine River	Minnesota	Mississippi River	46.70	−94.39	32	30	365	356	374	1.538	0.162	0.155	−0.031	22.610	2	NA	1
(21) Cut Foot Sioux	Minnesota	Mississippi River	47.50	−94.09	32	25	622	590	658	1.515	0.148	0.146	−0.017	22.730	2	NA	1
(22) Otter Tail Lake	Minnesota	Hudson Bay	46.41	−95.66	32	23	Infinite	Infinite	Infinite	1.554	0.158	0.156	−0.013	22.196	2	NA	2
(23) Red Lake	Minnesota	Hudson Bay	47.91	−95.04	32	29	103	102	104	1.506	0.153	0.148	−0.025	23.702	2	NA	2

Note

Columns include the population's state and major drainage of origin, latitude, longitude, number of individuals sampled, number of individuals missing genotypes at <20% of SNPs (*n* genotyped), effective population size (*N*_e_) estimates with lower and upper 95% confidence interval (CI), allelic richness (*A*_r_), observed (*H*_O_) and expected (*H*_E_) heterozygosity, *F*_IS_ estimates, and population groupings for the three hierarchical analyses of molecular variance (AMOVAs) performed. Populations excluded from an AMOVA are denoted by NA.

Regional patterns of population stratification were investigated using Monmonier's algorithm (Monmonier, 1973), implemented in adegenet v2.1.1 (Jombart & Ahmed, 2011), which can identify potential boundaries to geneflow. Upon constructing a spatial connectivity network among population geographic locations, performed here via Delaunay triangulation, Monmonier's algorithm identifies and intersects the strongest genetic distances between neighbors; genetic distances were calculated using Edwards’ (1971) Euclidean distance. To ensure that differentiation representative of genetic boundaries was not masked by random noise, genetic distances were scaled with a principal coordinate analysis in which only the coordinates corresponding to the first eigenvalue were retained (Figure S1). We performed three iterations of the Monmonier algorithm, as it converged on the first step of the fourth iteration when the default threshold for the minimum genetic distance that could be selected for path construction was applied. Having identified the presence of three major boundaries to gene flow, we reduced the threshold value by 5% to further evaluate isolation in regions of shallow structure.

We then examined population structure with two distinct individual‐based methods. First, we used admixture v1.3 (Alexander et al., 2009 #176), which provides individual‐based ancestry coefficients relative to a fixed number (*K*) of potential genetic groups. We used admixture to analyze three datasets, all sampled populations tested at *K* = 2 through *K* = 14 clusters, followed by hierarchical tests performed with only the populations sampled in Wisconsin (*K* = 2 through *K* = 10) and again using only populations sampled in Minnesota (*K* = 2 through *K* = 8). To examine support for each *K*, we applied fivefold cross‐validation to all tests.

Individual population structure was further investigated with fineRADstructure v0.3 (Malinsky et al., 2018), which explores the configuration of various sample stratifications by inferring a co‐ancestry matrix using a Bayesian approach, and selects the most probable configuration based on likelihood ratios between MCMC samples. This method is complementary to admixture, in that it leverages linkage among microhaplotypes to emphasize the most recent coalescence among individuals. Therefore, loci that successfully aligned to the yellow perch genome were arranged according to genomic position prior to conducting this analysis.

The individual co‐ancestry matrix was constructed in RADpainter (Malinsky et al., 2018) with default parameters, which then was used to construct population configurations through the *finestructure* MCMC algorithm (100,000 burn‐in iterations, 100,000 sampling iterations, and a thinning parameter of 1000). Finally, the maximum‐likelihood tree‐building algorithm (Lawson et al., 2012) was applied using 10,000 burn‐in iterations and default parameters. Intra‐basin and inter‐basin co‐ancestry coefficients were compared for the upper Chippewa and upper Wisconsin River basins using a Tukey's HSD test (*α* = 0.001) to determine whether significant differences in co‐ancestry existed in this region where considerable stocking effort has occurred.

### 2.4 Differentiation across the genome

To investigate genetic differentiation across the walleye genome, we first conducted three tests for loci displaying signals of divergent selection (outlier loci) with BAYESCAN v2.1 (Foll & Gaggiotti, 2008) using all loci, a conservative false discovery rate of 0.01, and the default parameters apart from prior odds, which we increased to 1000 to reduce false positives with a genomic dataset (default prior odds = 10). This analysis was conducted on three population groupings: (1) all populations, (2) populations from Minnesota, and (3) populations from Wisconsin. Next, we used a Gaussian kernel smoothing technique (Ackiss et al., 2020; Gagnaire et al., 2013; Hohenlohe et al., 2010) that incorporated locus‐specific differentiation and genomic position to identify highly differentiated genomic regions that may be undergoing divergent selection. A window size of 500,000 bp and a stepwise shift of 100,000 bp were used for this analysis, and values of genetic differentiation were weighted according to their window position as described by Gagnaire et al. (2013). This window size was chosen based on genome size and SNP density to target approximately 10 SNPs per window, a target which should provide good power for detecting divergent regions; our final results came very close to this number (mean of 10.7 SNPs per window). Highly differentiated windows were identified by randomly sampling *N* loci from the genome (where *N* was the number of loci in the window) and comparing the average differentiation of those loci to the average differentiation of the loci in the window. This sampling routine was conducted 1000 times for each window. If a window exceeded the 90th percentile of the sampling distribution, the number of bootstrap replicates was increased to 10,000. Contiguous windows that contained at least two loci and exceed the 99th percentile of the distribution after 10,000 bootstrap replicates were classified as significantly differentiated. We chose a minimum value of two loci per window to ensure that we could identify small but prolific windows. This analysis was conducted for the same three population groupings described above. We recognize that this sliding window analysis is relatively coarse due to the limited density of RAD data and does not take into account factors such as variation in recombination rate, which can influence the detection of highly differentiated regions (Burri et al., 2015). However, we believe the analysis is useful for identifying genomic regions that may be adaptively important that could be targets for future studies using high‐density approaches such as genome resequencing.

## 3 RESULTS

### 3.1 Sequencing and genetic diversity

Restriction site‐associated DNA sequencing data were available from Bootsma et al. (2020) for 954 walleye from 23 populations, with an average of 42 individuals per population. Sequencing yielded 1,463,269 retained reads per individual, on average (range = 22,506–8,156,976). After applying quality filters, 839 individuals (Table S1) and 22,675 SNPs (Table S2) were retained. A total of 18,006 of 22,675 (79%) RAD tags were successfully aligned to the yellow perch genome; 17,554 (98%) of the aligned RAD tags were also included in the microhaplotype dataset after filtering (Table S3). Genotyping rates across loci and individuals were slightly lower for the microhaplotype data (384 loci genotyped in 45% to 79% of individuals; 32 individuals genotyped at 76% to 80% of loci) than for the single‐SNP data (which required a minimum of >80% of genotyped loci or individuals), as a result of incorporating multi‐SNP genotypes. It was important, however, that we included the same loci and individuals in both single‐SNP and microhaplotype analyses.

Genetic diversity estimates were similar across sample locations, with an average *H*_E_ of 0.163 (0.143–0.176), *H*_O_ of 0.146 (0.141–0.184), and *A*_r_ of 1.58 (1.493–1.643) (Table 1). Estimates were, however, lower for Minnesota populations. For example, estimates of *H*_O_ were significantly lower in Minnesota populations (mean = 0.151, 0.143–0.163) than in Wisconsin populations (mean = 0.169, 0.159–0.176) when compared in a two sample *t*‐test (α = 0.05, *p*‐value <0.001). This difference in diversity may reflect true population differences, but could also be a function of ascertainment bias, as 15 populations from the dataset are from Wisconsin compared to eight for Minnesota. Estimates of *N*_e_ were in the hundreds for twelve populations, with estimates >1000 for nine populations and <100 for two populations, Sanford Lake (#9) and Lake Koronis (#18) (Table 1). Sanford Lake is the smallest lake in our study (37 ha), likely explaining the small *N*_e_. Lake Koronis is not a small lake but had the lowest sample size of any lake, which may have led to a lack of precisions when estimating *N*_e_. It is also possible that this low *N*_e_ reflects population declines due to habitat degradation as suggested by Minnesota Department of Natural Resources surveys, but analysis of additional samples would be needed to confirm this. There were no clear differences in *N*_e_ between Minnesota and Wisconsin populations.

### 3.2 Population structure

**FIGURE 2:**
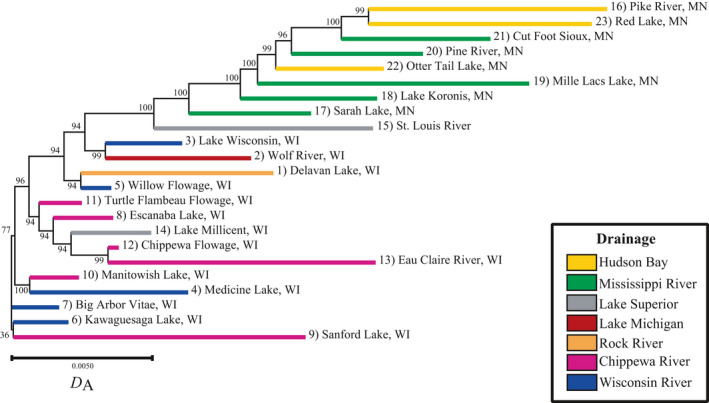
Neighbor‐joining dendrogram of walleye (*Sander vitreus*) populations sampled in Wisconsin (WI) and Minnesota (MN), USA. Nodes show bootstrap support, and branch lengths correspond to genetic distance estimated using Nei's *D*_A_. Branches are color coded according to the population's major drainage of origin (Hudson Bay: yellow; Mississippi River, MN: green; Lake Superior: gray; Chippewa River, WI: purple; Wisconsin River, WI: blue; Rock‐Fox River, WI: orange; Lake Michigan: red)

**FIGURE 3:**
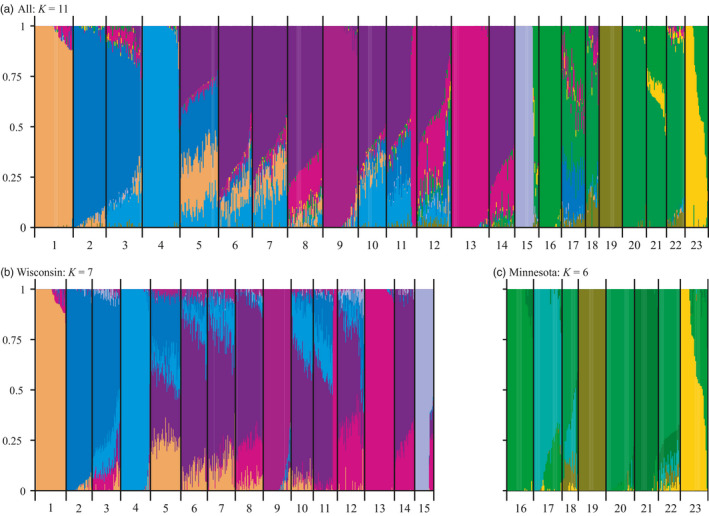
Admixture results for walleye (*Sander vitreus*), in Wisconsin and Minnesota, USA, showing individual ancestry proportions for three datasets: (a) all populations (*K* = 11), (b) only Wisconsin populations (*K* = 7), and (c) only Minnesota populations (*K* = 6). Individuals are grouped by population (labeled according to the population numbers in Table 1) and sorted in descending order relative to the predominant ancestry coefficient within the population. Pike River ancestry (#16, a known historical egg‐take source) was observed in migrants, originally stocked in the upper reaches of the St. Louis River (#15) and sampled in the lower portion, and in hybrids sampled in Red Lake, MN (#23). Multiple Wisconsin populations have served as egg‐take sources over time and many of the northern populations showed a signal of genetic homogenization, rather than a single source–sink relation

**FIGURE 4:**
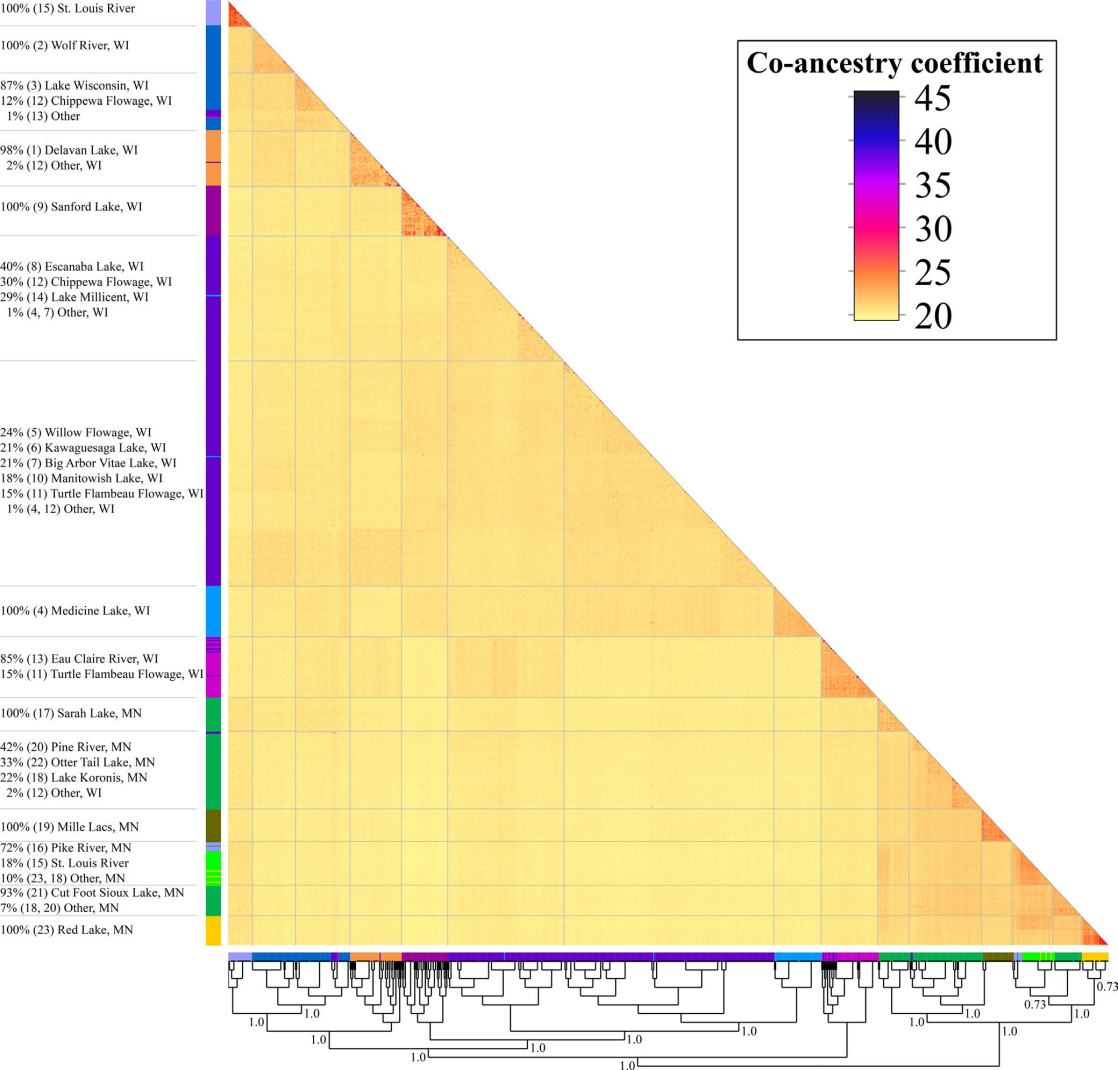
Matrix of pairwise co‐ancestry coefficients for all individuals successfully genotyped (higher values indicate stronger co‐ancestry) and the associated maximum‐likelihood tree from fineRADstructure. Individuals are labeled using color bars along each axis, with colors corresponding to the predominant ancestry of an individual's population within admixture (Figure 3). Gray lines divide relevant clades defined by major nodes within the tree, which have bootstrap support values indicated. The composition of populations within these groups is described on the left, with population numbers corresponding to those in Table 1. A list of specific individual identities is available in Table S5

The largest genetic differentiation in our dataset was between populations from Wisconsin and Minnesota (Figures 1, 2, 3, 4, Tables 2, 3). Interestingly, population structure did not necessarily coincide with major drainages. Low differentiation between populations in discrete drainages, as well as highly distinct populations that were dissimilar to other populations found in the same drainage, was observed. This was exemplified on a broad scale through AMOVAs, where a test between walleye sampled in Wisconsin and Minnesota indicated that an approximately equal degree of variation existed between states (3.13%) as was present among populations within the states (3.90%). Additionally, further tests performed using independent AMOVAs within each of the two states with populations grouped according to hydrogeography showed that a substantially higher amount of variation (~4%) was partitioned within groups (i.e., drainages) than among them (~0.5%) (Table 2).

**TABLE 2:** Results from three hierarchical analyses of molecular variance (AMOVAs) for walleye (*Sander vitreus*) in Wisconsin and Minnesota, USA

Source of variation	*df*	SSQ	Var	% of variation
All				
Among groups	1	39936.91	54.08	3.13
Within groups	21	137207.96	67.26	3.90
Within pops	1655	2656558.49	1605.17	92.97
Wisconsin				
Among groups	4	33994.29	10.51	0.61
Within groups	10	63073.48	55.18	3.18
Within pops	1241	2069472.47	1667.58	96.21
Minnesota				
Among groups	1	7049.11	7.05	0.47
Within groups	6	33091.07	78.32	5.21
Within pops	414	587086.02	1418.08	94.32

Note

The three AMOVAs were designed to test: (1) population structure between walleyes in Wisconsin and Minnesota, USA, (2) structure between hydrological basins in Wisconsin only, and (3) between hydrological basins in Minnesota only. The border water population, St. Louis River (population 15) was grouped with Wisconsin samples and excluded from the Minnesota AMOVA. Specific grouping within AMOVAs can be found in Table 1.

**TABLE 3:** Pairwise *F*_ST_ estimates for walleye *Sander vitreus* populations in Wisconsin (WI) and Minnesota (MN), USA

Population ID	1	2	3	4	5	6	7	8	9	10	11	12	13	14	15	16	17	18	19	20	21	22	23	
(1) Delavan Lake, WI																								
(2) Wolf River, WI	0.039																							
(3) Lake Wisconsin, WI	0.041	0.019																						
(4) Medicine Lake, WI	0.064	0.054	0.037																					
(5) Willow Flowage, WI	0.025	0.016	0.016	0.029																				
(6) Kawaguesaga Lake, WI	0.037	0.030	0.024	0.025	0.005																			
(7) Big Arbor Vitae Lake, WI	0.037	0.033	0.024	0.025	0.009	0*																		
(8) Escanaba Lake, WI	0.042	0.040	0.029	0.033	0.016	0.010	0.009																	
(9) Sanford Lake, WI	0.074	0.075	0.064	0.067	0.047	0.045	0.044	0.055																
(10) Manitowish Lake, WI	0.042	0.035	0.023	0.019	0.010	0*	0.003	0.009	0.047															
(11) Turtle Flambeau Flowage, WI	0.038	0.027	0.017	0.024	0.009	0.003	0.005	0.008	0.050	0.003														
(12) Chippewa Flowage, WI	0.037	0.033	0.020	0.031	0.016	0.009	0.011	0.008	0.057	0.009	0.004													
(13) Eau Claire River, WI	0.071	0.075	0.059	0.079	0.064	0.063	0.061	0.049	0.105	0.062	0.040	0.035												
(14) Lake Millicent, WI	0.047	0.047	0.030	0.038	0.019	0.005	0.009	0.008	0.059	0.009	0.007	0.006	0.046											
(15) St. Louis River	0.065	0.051	0.041	0.071	0.047	0.053	0.053	0.056	0.096	0.054	0.047	0.045	0.082	0.059										
(16) Pike River, MN	0.112	0.112	0.091	0.111	0.095	0.094	0.094	0.093	0.140	0.096	0.089	0.082	0.115	0.098	0.070									
(17) Sarah Lake, MN	0.060	0.054	0.037	0.061	0.043	0.044	0.044	0.042	0.089	0.044	0.036	0.030	0.063	0.044	0.043	0.053								
(18) Lake Koronis, MN	0.055	0.061	0.036	0.055	0.039	0.035	0.036	0.035	0.084	0.037	0.032	0.025	0.057	0.043	0.048	0.068	0.025							
(19) Mille Lacs, MN	0.094	0.098	0.076	0.096	0.078	0.077	0.077	0.076	0.122	0.078	0.073	0.066	0.098	0.083	0.082	0.097	0.060	0.050						
(20) Pine River, MN	0.080	0.080	0.061	0.081	0.064	0.064	0.062	0.060	0.109	0.064	0.057	0.049	0.082	0.064	0.058	0.062	0.038	0.024	0.057					
(21) Cut Foot Sioux, MN	0.081	0.087	0.061	0.083	0.065	0.064	0.064	0.063	0.111	0.066	0.061	0.052	0.085	0.072	0.054	0.049	0.039	0.042	0.070	0.032				
(22) Otter Tail Lake, MN	0.067	0.069	0.049	0.069	0.051	0.050	0.050	0.048	0.096	0.050	0.044	0.037	0.070	0.052	0.053	0.065	0.030	0.019	0.053	0.021	0.030			
(23) Red Lake, MN	0.103	0.104	0.084	0.105	0.088	0.088	0.088	0.087	0.133	0.089	0.083	0.076	0.108	0.093	0.076	0.068	0.064	0.068	0.097	0.071	0.055	0.064		

Note

Sample locations are numbered according to Table 1. Low *F*_ST_ values are shaded in white, while high *F*_ST_ values are shaded dark gray. All values are significant (*p* < 0.001) unless denoted by *.

Patterns of genetic divergence between Wisconsin and Minnesota samples closely mirrored patterns of recolonization hypothesized by Billington (1996). The first boundary identified by Monmonier's algorithm, indicating the greatest genetic distance among populations, was identified between populations from Wisconsin and Minnesota (Figure 1b). This divergence was further supported by the dendrogram, where Wisconsin populations formed generally shallow clades that grouped away from Minnesota populations, which all exhibited long branches (Figure 2). Additionally, pairwise *F*_ST_ values (Table 3) were higher for Wisconsin to Minnesota comparisons (0.07 on average) than either within‐Minnesota comparisons (0.05 on average) or within‐Wisconsin comparisons (0.03). Finally, the pattern of high differentiation between Minnesota and Wisconsin populations was also evident on an individual‐based level, as admixture and fineRADstructure results showed negligible amounts of overlap in co‐ancestry between individuals from the two states (Figures 3 and 4).

Initial admixture results obtained while testing co‐ancestry of all samples indicated *K* = 9 as the most parsimonious number of distinct genetic groups in our full dataset (Figure S2). Additional structure was, however, clearly visible at *K* = 11 within this dataset and only displayed a marginal increase in cross‐validation error compared to *K* = 9 (Figure S3). Moreover, this structure was well supported by analysis of the dendrogram (Figure 2) and fineRADstructure (Figure 4). Therefore, the full dataset is presented for *K* = 11 (Figure 3a) but see *K* = 9 in the Figure S2; See Figure S4 for all tested values of *K*. The 11 clusters were somewhat associated with major drainages, but there also were frequent deviations from this pattern. Some examples of population structure aligning with drainage boundaries include Delevan Lake (#1), which is the only population in our dataset from the Rock River drainage and was genetically unique, and the St. Louis River (#15), which is found in the Lake Superior drainage and was also genetically unique.

Within‐state comparisons revealed higher differentiation among samples from Minnesota (mean pairwise *F*_ST_ = 0.053, 0.019–0.097) than that observed among samples from Wisconsin (mean pairwise *F*_ST_ = 0.036, 0.000–0.105), on average (Table 3). It was also evident from the dendrogram that walleye populations sampled in Minnesota (populations #16–23) displayed elevated genetic structure in relation to Wisconsin (#1–15) populations, as branches were generally longer in the Minnesota portion of the tree (Figure 2). This higher divergence within Minnesota populations was also evident in the fineRADstructure results, where intra‐population co‐ancestry values were significantly higher for Minnesota populations (mean intra‐population co‐ancestry = 22.827) than Wisconsin populations (mean intra‐population co‐ancestry = 21.910, Figure 4) when compared with a two sample *t*‐test (*α* = 0.05, *p*‐value = 0.016).

Within Minnesota, populations generally grouped by drainage according to the dendrogram, admixture analysis, and fineRADstructure analysis (Figures 2, 3, 4). The largest exception to this was Otter Tail Lake (#22), which clustered more closely to upper Mississippi River populations than to other Hudson Bay drainage populations. Substructure was also present within the Hudson Bay and Mississippi river drainages, as evidenced by relatively high intra‐population co‐ancestry values in fineRADstructure (Figure 4) and long branches on the dendrogram (Figure 2). Interestingly, hierarchical admixture analysis including samples only from Minnesota revealed additional genetic structure that was not visible with the full admixture plot. Specifically, at *K* = 6, Sarah Lake (#17) and Cut Foot Sioux Lake (#21) appeared to represent largely distinct genetic clusters (Figure 3c), whereas they appeared potentially admixed in the overall admixture plot (Figure 3a). Therefore, *K* = 6 was selected as the most informative admixture result to visualize for the Minnesota dataset as it had similar cross‐validation errors as other *K* values (Figure S3) and produced the most biologically relevant representation of genetic structure based on the maximum‐likelihood tree from fineRADstructure. The most notable evidence of historical stocking in Minnesota was in Red Lake (#23), which was highly admixed and composed of putative native walleye (Figure 3, yellow bars), fish of Pike River origin (#16, neon green bars), and putative hybrids. Samples from the St. Louis River (#15) also showed evidence of individuals from the Pike River (#16) population, with admixture showing 6 of the 30 individuals genotyped having a high degree of co‐ancestry (59%–80%) with individuals from Pike River (Figure 3a).

Our results suggest that the St. Louis River represents a unique genetic group likely consisting of native Lake Superior ancestry that was not present elsewhere in our other samples. The second boundary identified via Monmonier's algorithm subdivided the St. Louis River (#15), a population on the border of Minnesota and Wisconsin in the Lake Superior watershed, and the rest of the populations in Wisconsin. Isolation between the St. Louis River and all other sampled populations was supported by admixture (Figure 3a) in which samples primarily displayed a unique ancestry with little to no admixing (see discussion for putative migrants, i.e., green bars); it was also supported by the dendrogram (Figure 2), where the St. Louis River was effectively equidistant from the nearest Minnesota sample (*D*_A_ = 0.013) as it was from the nearest Wisconsin sample (*D*_A_ = 0.014). In contrast to this signal of unique ancestry for the St. Louis River, Monmonier's algorithm, the dendrogram, admixture, and fineRADstructure results indicated that the other population sampled in the Lake Superior watershed, Lake Millicent (#14), possessed ancestry similar to ancestry observed in the upper Chippewa basin (#8–12), which is consistent with stocking records indicating that this population was founded from broodstock collected in the upper Chippewa basin (Jeff Scheirer, Wisconsin Department of Natural Resources, personal communication; Figures 1, 2, 3, 4).

Although populations in Wisconsin generally displayed lower genetic structure than those in Minnesota, a subdivision between the lower reaches of the Wisconsin and Chippewa rivers in Wisconsin (Figure 1) was identified by the third boundary produced using Monmonier's algorithm. The Eau Claire River (#13) in the lower Chippewa drainage was highly distinct from most populations in the dataset, with some shared ancestry with the Chippewa Flowage (#12) but had little shared ancestry with upper Chippewa populations (Figure 3). Additionally, the Lake Wisconsin (#3) population was more similar to the geographically proximate population from the Wolf River (#2), which is in the Lake Michigan drainage, than it was to upriver populations in the Wisconsin River. Despite the apparent shared ancestry between the Lake Wisconsin and Wolf River populations, these populations were distinguished with 100% accuracy by the fineRADstructure neighbor‐joining tree. Although these populations may have a shared evolutionary history with respect to postglacial recolonization, they have since diverged from one another (Figure 4).

The genetic distinctness of populations in the lower Wisconsin and Chippewa rivers sharply contrasts the genetic similarities observed in the most upriver populations from this region. For many of these upriver populations, a single genetic background represented a large proportion of many individual's ancestry in admixture results (Figure 3). These populations also displayed relatively short branches in the dendrogram and low pairwise *F*_ST_ values (Figure 2, Table 3); the most evident examples of this pattern were Kawaguesaga Lake (#6), Big Arbor Vitae Lake (#7), Manitowish Lake (#10), and the Turtle Flambeau Flowage (#11). Analysis in fineRADstructure (Figure 4), however, did reveal sub‐structuring consistent with geographic connectivity. The average co‐ancestry coefficient was significantly higher (*p* < 0.001) for intra‐basin comparisons in this region than were inter‐basin comparisons. Furthermore, the neighbor‐joining tree constructed as part of this analysis identified two large groups whose configuration generally reflected the geographic distribution of individuals throughout this region (Figure 4). The first of these two groups consisted almost exclusively of individuals derived from sources originating in the upper Chippewa River (99% from #8, #12, or #14), while the majority of the second group consisted predominantly of individuals from the upper Wisconsin River (66% from #5, #6, or #7). In comparing co‐ancestry coefficients for individuals in these headwater populations relative to individuals from the mainstem population in the Chippewa River (Eau Claire River, #13, pink individuals), it was evident that the first group had a higher degree of co‐ancestry with Chippewa populations than the second group, which was more closely associated with upper Wisconsin River populations. However, two Chippewa basin populations, Manitowish Lake (#10) and the Turtle Flambeau Flowage (#11), did group more closely with upper Wisconsin populations. Despite this, most individuals from Manitowish Lake and Turtle Flambeau Flowage grouped within a separate sub‐node from the other Wisconsin River populations.

Although populations in the upper Wisconsin and Chippewa rivers were generally similar, there were a few notable exceptions. The first of these was Sanford Lake (#9), which was highly distinct in all analyses. This population had the lowest *N*_e_ in our study (*N*_e_ = 44, Table 1) and may have been subject to the effect of genetic drift from other neighboring populations. The next highly differentiated population in this region was Medicine Lake (#4). This population has relatively high genetic diversity, but may have experienced historical stocking from out‐of‐basin sources. Finally, admixture analysis indicated that the Willow Flowage (#5) represents an admixture of multiple genetic clusters corresponding to Wolf River, Rock River, and upper Wisconsin River ancestry (Figure 3); this reservoir population was likely established when the dam was built in 1926 and may have been founded from or stocked with fish from all three of these genetic groups.

### 3.3 Differentiation across the genome

**FIGURE 5:**
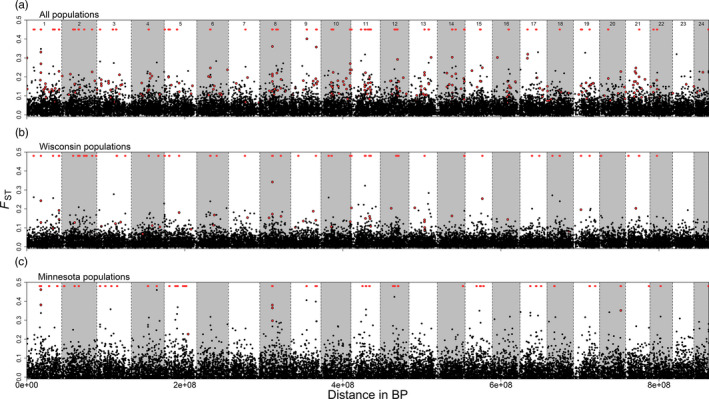
Genetic differentiation for walleye loci aligned to the yellow perch genome across three datasets. Each black dot represents a marker, red dots are putative outliers according to BAYESCAN analysis, and red lines denote significantly differentiated windows identified with kernel smoothing analysis. Chromosomes are separated by dashed lines. See Table S2 for information on each marker

In general, outlier loci and highly differentiated genomic regions were spread throughout the genome, with few notable areas of high divergence. BAYESCAN analysis identified 341 putative outlier loci in the all population dataset, 55 putative outliers in the Wisconsin population dataset, and nine putative outliers in the Minnesota population dataset (Figure 5, Table S2). Three of these loci were outliers in all three comparisons, 48 loci were outliers in two of the three comparisons, and four were outliers in Minnesota and Wisconsin comparisons separately, but not in the full dataset. Alignments to the yellow perch genome were possible for 280 of 349 total outlier loci. At least three outlier loci were found on each chromosome, with an average of 12 outliers per chromosome (range 3–24). Examination of allele frequencies for the highest *F*_ST_ outliers did not reveal any consistent patterns, and many of the high *F*_ST_ loci were fixed or had low minor allele frequencies in most populations, with a few populations that were more variable. It is important to note that while type 1 error is generally lower with BAYESCAN than other similar programs (Narum & Hess, 2011), BAYESCAN assumes populations follow a simple island model, which is violated in our dataset. This is especially true for the full dataset that likely includes populations from different glacial refugia, which may have led to some false positives (see Lotterhos & Whitlock, 2015; De Villemereuil et al., 2014). Nevertheless, results from BAYESCAN are useful for highlighting highly differentiated markers in our dataset that may be undergoing positive selection.

In total, 154 highly differentiated genomic regions were found in the all population dataset, 126 regions were found in the Wisconsin population dataset, and 126 regions were found in the Minnesota population dataset (Figure 5). Twelve of these regions were found to be significantly differentiated in all three datasets, 13 were significantly differentiated in Minnesota and Wisconsin datasets, and 71 regions were significantly differentiated in two of the three datasets. Highly differentiated windows were found all on chromosomes, with an average of 14 windows per chromosome and a range of two to 29 windows. Although few genomic regions were differentiated in all three comparisons, we did discover an approximately 1‐MB area of chromosome 8 that contained six differentiated windows common to all three comparisons, and an approximately 0.8‐MB area of chromosome 11 that contained four differentiated windows common to all three comparisons. We extracted the annotation features table from the yellow perch genome for these two regions and discovered some interesting genes (Table S4). The differentiation region on chromosome 8 contained recombination activating (RAG) genes, which play an important role in immune systems function and have been the target of evolutionary genetic studies for decades (e.g., Brandle et al., 1992), and the region on chromosome 11 contained zinc finger proteins, which have been shown to differentially expressed between different life history types of salmon (McKinney et al., 2015). While caution must be taken when linking annotations to results from genome scans (Pavlidis et al., 2012), these regions certainly represent interesting targets for future study.

## 4 DISCUSSION

As anticipated (Allendorf et al., 2010), the transition from genetics to genomics has provided increased precision for elucidating neutral population structure (e.g., Emerson et al., 2010) and investigating evolutionary processes that were difficult to untangle with classical genetic methods (e.g., Therkildsen et al., 2019). Our study represents an example of how genomic methods can be leveraged to understand complex patterns of population structure in a commercially, culturally, and recreationally important freshwater fish with an extensive history of stocking. Using GBS data, we document genetic divergence among geographically isolated walleye populations throughout Wisconsin and Minnesota and shifts in the genetic composition of populations, apparently due to stocking, at the finest resolution to date. Although genomic techniques have proven valuable for testing for the effects of stocking on the genetic structure of fish populations (Bay et al., 2019; Kovach et al., 2016; Létourneau et al., 2018), they have rarely been used for this purpose outside of salmonids. Furthermore, nonsalmonid, freshwater fish species that have been studied in this context have typically been characterized by high differentiation (Brauer et al., 2018; Rougemont et al., 2019). In contrast, we observed relatively low levels of differentiation between populations, In contrast, we observed relatively low levels of differentiation between populations, on a scale only slightly higher than highly migratory, marine species (Ward, 2006). Nevertheless, we were still able to disentangle complex patterns of population structure using recently developed genomic tools. In particular, the application of fineRADstructure, which leverages microhaplotype data and analysis of most recent coalescence among individuals, helped us to identify the presence of fine‐scale genetic structure including structure among tributaries to the Mississippi River with extensive histories of stocking from nonlocal sources. Our study highlights the utility of pairing genomic data with newly developed analytical methods to clarify complex patterns of population structure, an approach that will be highly applicable to many species and systems.

### 4.1 Disentangling complex patterns of population structure

Our analyses revealed complex patterns of genetic structure for walleye in Wisconsin and Minnesota that were likely primarily driven by recolonization of different lineages following deglaciation and through anthropogenically facilitated gene flow through stocking. Our study region was recolonized by multiple lineages of walleye after relatively recent glacial recession (~10,000 years ago; Syverson & Colgan, 2011). At the close of the Pleistocene Epoch, the Laurentide Ice Sheet underwent several episodes of retreating and readvancing. During this time, several glacial lakes, whose volume and size were related to the movements of the ice margin, were formed in our study region (Larson & Schaetzl, 2001; Wright, 1990): the southern part of the Michigan basin, which presumably drained south into the Mississippi drainage, the western arm of the Superior basin, which drained south through the St. Croix River valley into the Mississippi basin, and the largest glacial lake, Lake Agassiz which formed in the Red River basin and naturally drained north toward Hudson Bay. When the ice sheet readvanced, however, north draining rivers were blocked, forcing Lake Agassiz to drain southward toward the Mississippi River basin (Mann et al., 1999). As the ice sheet fully receded and the lakes drained, these southern outlets were eventually blocked by watershed divides that created current drainage boundaries with the Great Lakes draining through the St. Lawrence River and the Red River basin again draining north to Hudson Bay.

The deep population divergences in our study (i.e., differences between Minnesota and Wisconsin populations) likely correspond to genetic differences between the ancient lineages that recolonized the landscape postglaciation (Billington, 1996; Billington et al., 1992). Because few samples were collected directly on the border between states, further sampling in the region would help to more precisely identify the genetic boundary between lineages. Population divergence within these major lineages is, however, generally lower for walleye than for other species within the region that have been studied. For example, microsatellite studies in species that also colonized the region relatively recently such as smallmouth bass (*Micropterus dolomieu*; Euclide et al., 2020), rock bass (*Ambloplites rupestris*; Westbrook, 2012), johnny darter (*Etheostomoa nigrum*; Westbrook, 2012), and muskellunge (*Esox masquinongy*; Turnquist et al., 2017) have revealed relatively high divergence across similar geographic scales. It is likely that this lower divergence in walleye is a function of larger population sizes (especially compared to muskellunge), higher rates of natural gene flow due to the more vagile nature of walleye, and higher rates of anthropogenically induced gene flow due to stocking.

One genetic pattern of note that we observed was the large differences between upriver and lower river populations in Wisconsin. Specifically, the Lake Wisconsin population (#3) was more similar to the Wolf River population (#2) than to upriver populations in the same watershed, and the Eau Claire River population (#9) was different from all other populations in the study including upriver populations in the same watershed (Figures 1a, 3 and 4). We postulate that these differences may indicate that lower river populations were colonized from different sources than upriver populations and that drainage boundaries have since shifted. For example, although Lake Wisconsin is in the Mississippi drainage, it is geographically proximate to the Wolf River drainage, which is in the Lake Michigan drainage. There is a low‐lying area near Portage, WI where the drainages are only separated by a few km. It is possible that the walleye population from Lake Wisconsin was colonized by the same source as the Wolf River population before the drainage patterns changed. A similar scenario may have occurred for the Eau Claire River population; however, we did not screen samples from nearby areas (e.g., the St. Croix River) and are therefore unable to test this hypothesis. Future sampling between the headwaters and mouths of these systems could provide additional insight into these differences, as well as the underlying mechanisms.

Although we believe the differences between upriver and downriver populations were not caused by stocking, there are many examples of altered population structure due to stocking in our dataset. The most conspicuous example of this pattern is in the upper Wisconsin and upper Chippewa watersheds; populations from these two watersheds are separated by about 1000 km of riverine habitat, but are geographically proximal over land, with <50 km between some populations that straddle the drainage boundary. Historically, many lakes near this watershed boundary have been stocked with fish from Art Oehmcke State Fish Hatchery (blue triangle in Figure 1a), which collects broodstock from lakes in the Wisconsin River drainage, resulting in cross‐basin stocking for populations in the upper Chippewa (personal communication, Steve Gilbert, Wisconsin Department of Natural Resources). This cross‐basin stocking has likely led to genetic homogenization (i.e., loss of genetic integrity) among populations in the region, a pattern that was observed in our study as well as a previous study using microsatellites (Hammen & Sloss, 2019). One alternative explanation for this pattern is changes in drainage patterns due to stream capture, but results from rock bass and johnny darter, two nongame species with no history of stocking, showed significant genetic differentiation across the boundary, indicating that stocking is likely the cause of genetic homogenization (Westbrook, 2012).

The convoluted stocking history of the upper Wisconsin/upper Chippewa region makes disentangling patterns of genetic structure difficult, but genomic data were able to clarify several patterns that were ambiguous based on microsatellites. In general, patterns of population structure were similar based on the two marker types, with our study and Hammen and Sloss (2019) providing support for small but detectable genetic differentiation between Chippewa River and Wisconsin River populations (Table 3) and indicating that populations from the Eagle River Chain (e.g., Medicine Lake #4) are genetic outliers and were likely founded from out‐of‐basin or bottlenecked sources (Figures 2 and 3). Genomic data were, however, able to substantially clarify relationships for the Willow Flowage (#5) and Lake Millicent (#14). Microsatellite data suggested that Willow Flowage and Lake Millicent populations were genetically similar and possibly of the same genetic lineage, while our data clarified that Lake Millicent was founded with individuals of Chippewa River origin and Willow Flowage likely was founded by multiple stockings encompassing different genetic ancestries (Figure 3). Finally, microsatellite data suggested that Kawaguesaga Lake (#6) in the Wisconsin River drainage and the Turtle Flambeau Flowage (#11) in the Chippewa River drainage were genetically indistinguishable, whereas our fineRADstructure analysis was able to elucidate slight differences between these populations (Figure 4, Table S5). Our finding indicates that populations that have been heavily stocked with out‐of‐basin fish, such as the Turtle Flambeau Flowage, may still retain some native alleles.

Evidence of stocking also existed in populations from Minnesota, most notably Red Lake (#23). The Red Lake population, which was historically used as an egg‐take source for stocking programs, experienced substantial declines in abundance due to overexploitation (Gangl & Pereira, 2003), resulting in closure of egg‐take operations in 1979, commercial fishing in 1997, and sport fishing in 1998. Through suspension of commercial and recreational fishing and the repeated stocking of fry from the genetically divergent Pike River (#16) population, the Red Lake walleye population was able to recover, and the fisheries were reopened in 2006 (Logsdon et al., 2016). Although the large‐scale stocking of Pike River walleye supported the recovery of Red Lake walleye, this stocking effort has substantially influenced the genetic composition of the native population. After the cessation of stocking in 2003, Logsdon et al. (2016) observed about 50% Pike River ancestry present in the 2007 year‐class based on microsatellite data, suggesting that a high degree of introgression had occurred. Our analysis of the 2017 year‐class indicates that Red Lake was composed of roughly 1/3 fish of pure Red Lake ancestry and 2/3 Pike River/Red Lake hybrids, with an average Pike River ancestry of 31% (Figure 3), suggesting that Pike River ancestry has decreased over time.

The only other population from Minnesota that displayed obvious evidence of stocking from nonlocal sources was the St. Louis River (#15). In this population, seven individuals had genetic ancestry consistent with stocking from Pike River (#16), 22 displayed putative Lake Superior ancestry, and no putative hybrids were observed (Figures 3 and 4). Pike River is a primary source for stocked walleye in Minnesota and individuals from this strain are stocked into the St. Louis River above multiple dams where the risk of individuals mixing with native fish in the lower river was thought to be low. Nevertheless, our data suggest that Pike River walleye likely travel over these dams, creating a potential genetic hazard of outbreeding depression for native populations downriver.

Although influences of undocumented historical stocking cannot definitively be excluded, the greater similarity of the Otter Tail Lake population to Mississippi headwaters populations instead of the Red Lake within the same basin may be due to different postglacial colonization patterns. Glacial Lake Agassiz, which covered much of the current Red River basin, once drained south via the Minnesota River valley to the Mississippi River (Wright, 1990), providing connectivity between the basins. The headwaters of the Minnesota River and the north‐flowing Red River are now separated by a short distance in western Minnesota. Otter Tail Lake itself is close to a tributary to the Minnesota River, and Radke (1992) proposed that the Otter Tail River was captured by the Red River after Lake Agassiz drained. Similar to our results, Mccusker et al. (2014) explained that pugnose shiner (*Notropis anogenus*) populations in the region were genetically closer based on geography rather than watershed barriers.

### 4.2 Microhaplotypes provide a valuable tool for increasing resolution of population structure

Since the days of allozymes, population geneticists have been searching for better ways to resolve subtle and convoluted population structure (Triantafillos & Adams, 2001). The genomics revolution promised to drastically increase resolution of population structure and has largely delivered (Bernatchez et al., 2017). However, one area of research that was highly touted (Allendorf et al., 2010; Funk et al., 2012) but has not necessarily delivered the results to match its lofty projections is the use of adaptive loci to disentangle population structure and define conservation units (Shafer et al., 2015). Although there have been a handful of notable studies that have identified large‐effect loci of adaptive significance and proposed ways to integrate knowledge of these loci into conservation strategies (Pearse et al., 2014; Prince et al., 2017), these findings are relatively rare and it is unclear whether conserving populations based on genotypes at major effect loci will lead to improved population viability (Kardos & Shafer, 2018, Waples & Lindley, 2018). More typically population genomic studies that conduct genome scans find that structure at outlier loci generally mirrors that at neutral loci (e.g., Batista et al., 2016; Garcia et al., 2020; Moore et al., 2014). Our data match this trend, with outlier loci generally spread across the genome and similar patterns of genetic structure revealed by putatively neutral and putatively adaptive loci (data not shown). Additionally, although the identification and use of high *F*_ST_ outlier loci to differentiate closely related populations has provided increased resolution in some systems such as for sockeye salmon (*Oncorhynchus nerka*) populations in Alaska (Ackerman et al., 2011), the dangers associated with high‐grading bias have decreased the use of this approach (Anderson, 2010; Waples, 2010). Instead, many studies with the goal of differentiating closely related populations are adopting a “brute‐force” approach that leverages data from large numbers of mostly neutral loci to disentangle complex relationships (Dussex et al., 2018; Mckinney et al., 2019; Silliman, 2019).

Genomic technology facilitates efficient construction of datasets containing thousands of loci for any organism, meaning that researchers are largely no longer data‐limited (Shafer et al., 2015). Many current analytical approaches (e.g., Arlequin; Excoffier & Lischer, 2010) were, however, designed for nongenomic markers such as microsatellites, and may not be able to take full advantage of the characteristics of genomic data. As the field of genomics has matured, it has become clear that simply genotyping more markers and analyzing them with traditional approaches has diminishing value for many conservation genomic studies (e.g., Nelson & Anderson, 2013), and that the most substantial advances may come from developing or adopting new approaches to analyze genomic datasets. One such recent advance is the use of microhaplotype markers, which has been shown to provide improved accuracy for inference of population structure (Lavretsky et al., 2019; Leitwein et al., 2020; Mckinney, Seeb et al., 2017; Rougemont et al., 2019). Analysis of microhaplotype data in fineRADstructure allowed us to identify subtle structure between populations that was not found with microsatellites or other SNP‐based analyses. Our data illustrate that microhaplotypes can capture signals of genetic structure that are not observable with other approaches, and we suggest that future studies incorporate haplotype information to improve resolution of complex population structure.

### 4.3 Management implications and conclusions

Our data suggest that most walleye populations throughout Minnesota and Wisconsin have largely retained their genetic ancestry, but that stocking has substantially altered population structure and reduced genetic integrity in some areas (c.f. Marie et al., 2010). Altered population structure is potentially a cause for concern, as introgression from stocking out‐of‐basin sources can reduce biocomplexity (Williamson & May, 2005), introduce selectively disadvantageous alleles (Hansen et al., 2010), and lead to the loss of rare alleles and coadapted gene complexes (Kitada et al., 2009). All populations in our study did, however, appear to harbor at least some native ancestry, and the degree of non‐native ancestry in the heavily stocked Red Lake population appears to be decreasing, suggesting that walleye populations may be able to purge non‐native alleles over time. This reduction in non‐native ancestry over time is encouraging and consistent with the hypothesis that the effects of stocking are potentially reversible through selection or drift, as suggested by other studies (Kovach et al., 2016; Létourneau et al., 2018; Perrier et al., 2013; Rougemont et al., 2019; Valiquette et al., 2014).

We suggest adopting genetically cognizant stocking protocols (Fisch et al., 2015; Miller & Kapuscinski, 2003) for walleye in Minnesota and Wisconsin to minimize stocking across genetic units where stocked walleye may influence naturally reproducing populations through direct supplementation or unimpeded connectivity. Although our study did not evaluate the fitness consequences of admixing strains, the persistence of native ancestry in many populations despite widespread stocking suggests a fitness advantage conferred by local ancestry. Each state has currently adopted the following management units: Rock/Fox, Lake Michigan, and upper Mississippi for Wisconsin; Mississippi headwaters, Red River, Rainy River (Pike River) and Superior for Minnesota. Based on our data, we suggest a re‐evaluation of genetic management units for Wisconsin that includes the definition of separate units for the upper Chippewa and upper Wisconsin and the adoption of different units for upriver and lower river populations. For Minnesota, we suggest maintaining current management units, which focus on northern Minnesota populations, and reassessing the current policy that has no restrictions on sources used for stocking in southern Minnesota. Sarah Lake has an introduced population that microsatellite data suggest may have originated from southern Minnesota (L. Miller, Minnesota Department of Natural Resources, *unpublished data*); its sample forms a distinct cluster in admixture and fineRADstructure analyses, suggesting that potential remnant populations in southern Minnesota that may warrant their own genetic management unit. We also suggest that additional studies could be conducted to refine estimates of genetic structure by increasing sample resolution in areas such as the mainstem Mississippi and St. Croix watersheds, which were not represented in our study, and in the Red River headwaters and nearby Mississippi basin to further resolve relationships near this major watershed boundary.

In conclusion, we leveraged genomic data and recently developed analytical approaches to disentangle complex patterns of population structure in walleye from Minnesota and Wisconsin. Genetic differentiation was high between the states, likely due to recolonization from different Pleistocene lineages, but differentiation within states (especially Wisconsin) was lower. We documented substantial evidence of stocking, including genetic homogenization of populations in the upper Wisconsin and Chippewa river drainages and hybridization between native and non‐native individuals in Red Lake. Our resolution for defining genetic structure was greater than that of previous microsatellite studies, and microhaplotype analysis allowed us to identify subtle genetic structure that was not present with microsatellite or single‐SNP analyses. Our study is one of the first to test for the influence of stocking on genetic structure outside of salmonids and outlines an approach for disentangling complex population structure that will be highly applicable to other species and systems.

## CONFLICT OF INTEREST

The authors declare no conflicts of interest.

## Supporting information

Table S1‐S5

Fig S1

Fig S2

Fig S3

Fig S4

## Data Availability

Raw data for the RADseq data used in this study were deposited to the NCBI sequence read archive (Accession: PRJNA655229), and VCF files of genotypes are available on DRYAD (https://doi.org/10.5061/dryad.h9w0vt4g0).
