# Supplementary figures and images for: The ghosts of propagation past: haplotype information clarifies the relative influence of stocking history and phylogeographic processes on contemporary population structure of walleye (Sander vitreus)
Source: Evol Appl. 2021 Jan 29;14(4):1124–44. doi: 10.1111/eva.13186 (PMC8061267; doi:10.1111/eva.13186)

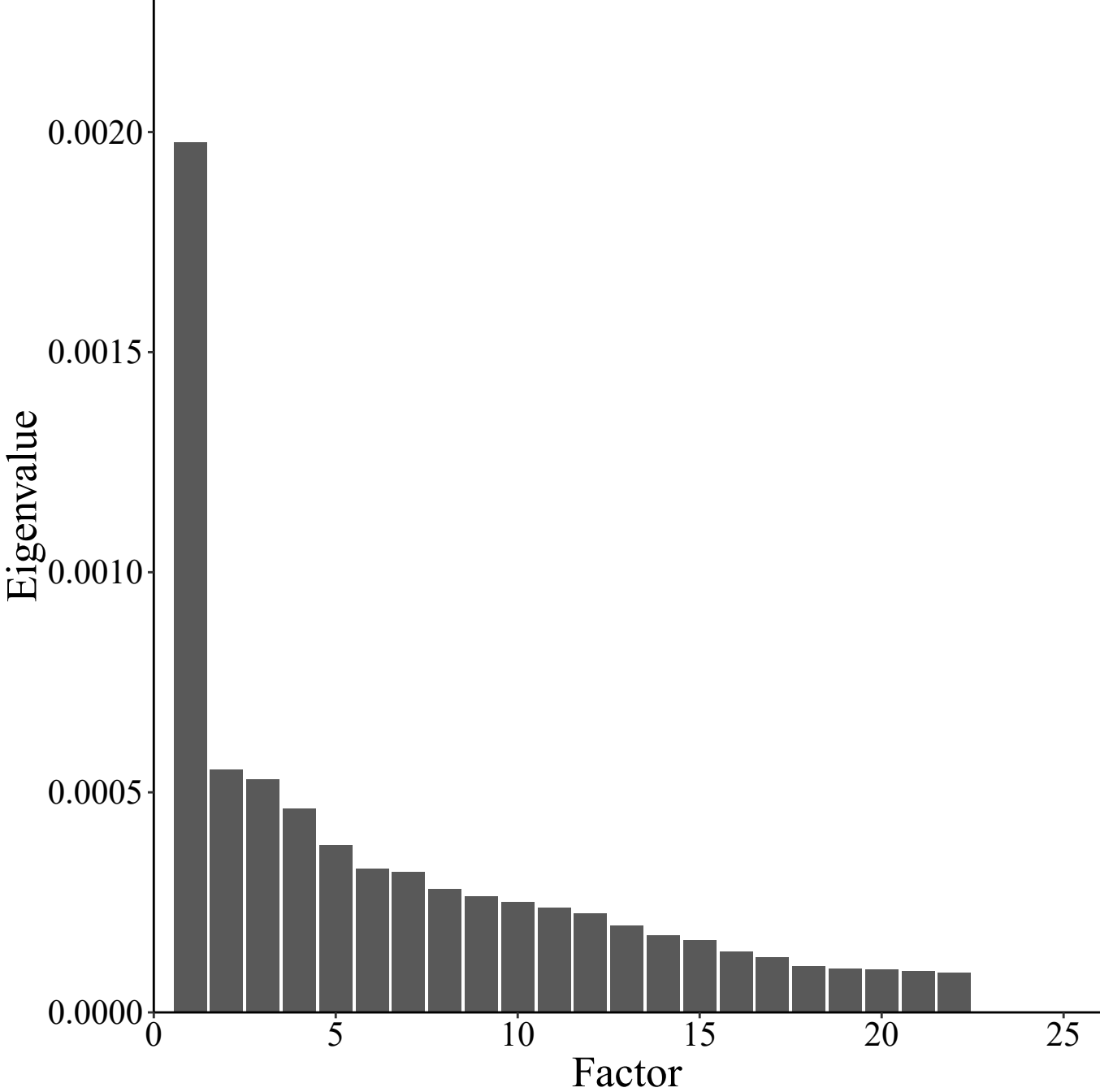

Supplement: Supplementary file 2 — Fig S1 [file EVA-14-1124-s003.pdf]

All:  $K = 9$

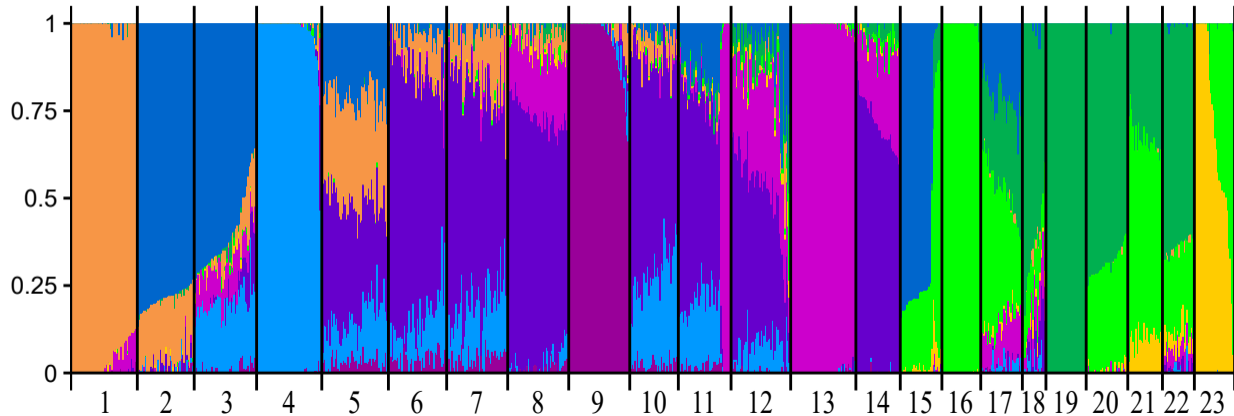

Supplement: Supplementary file 3 — Fig S2 [file EVA-14-1124-s004.pdf]

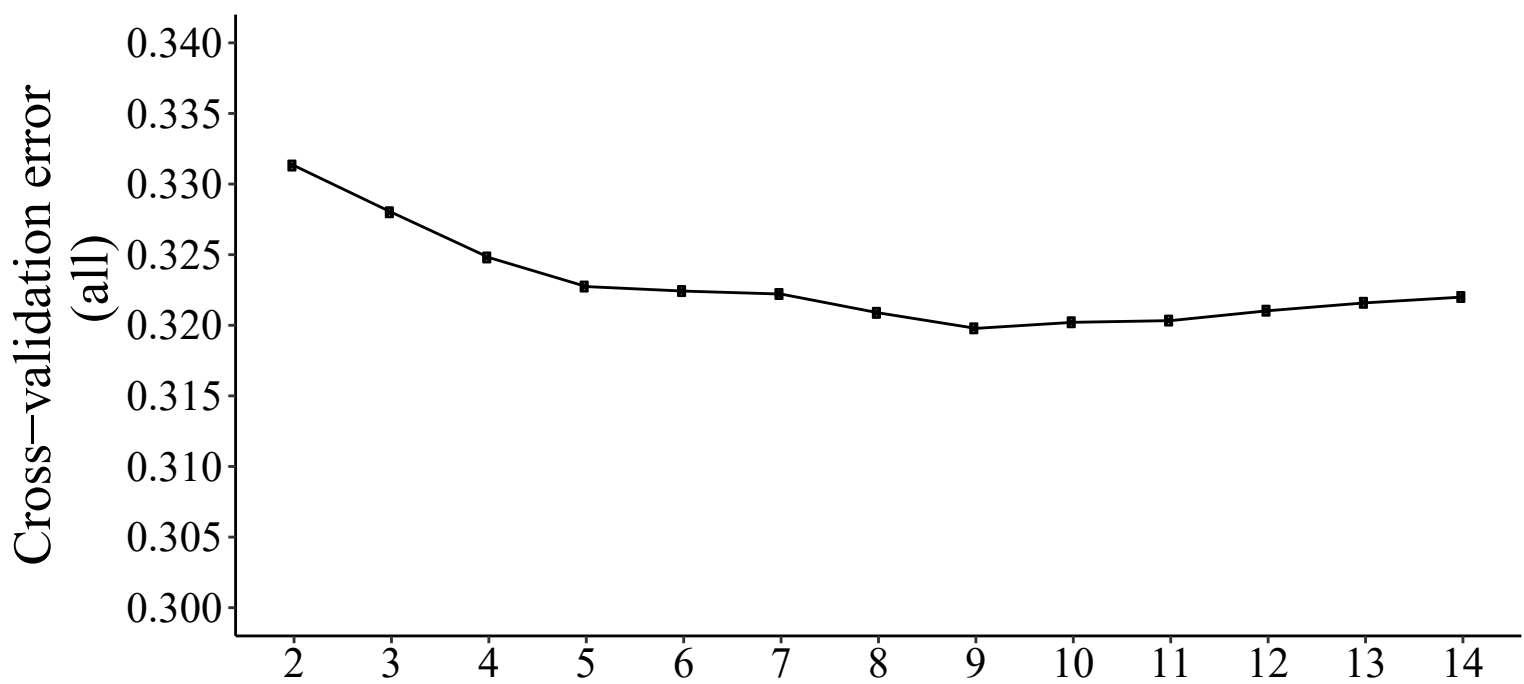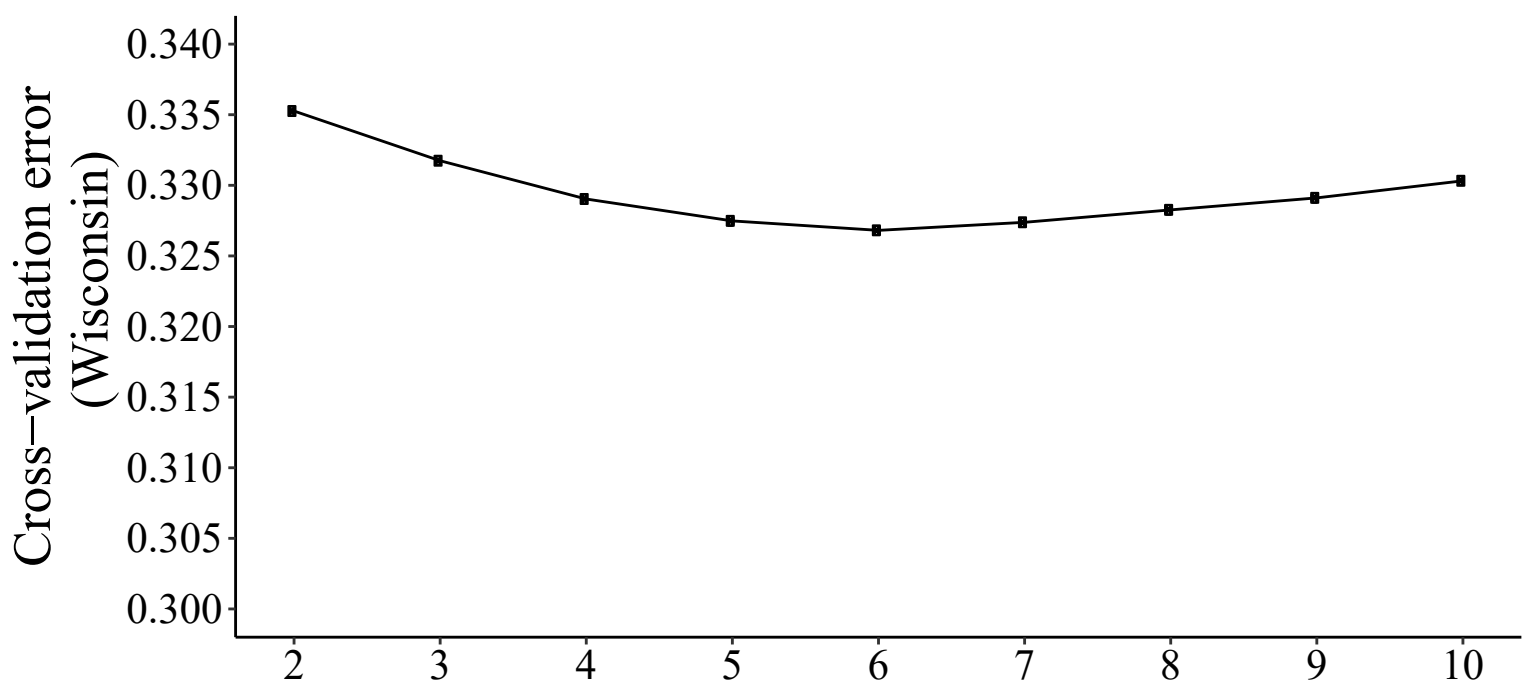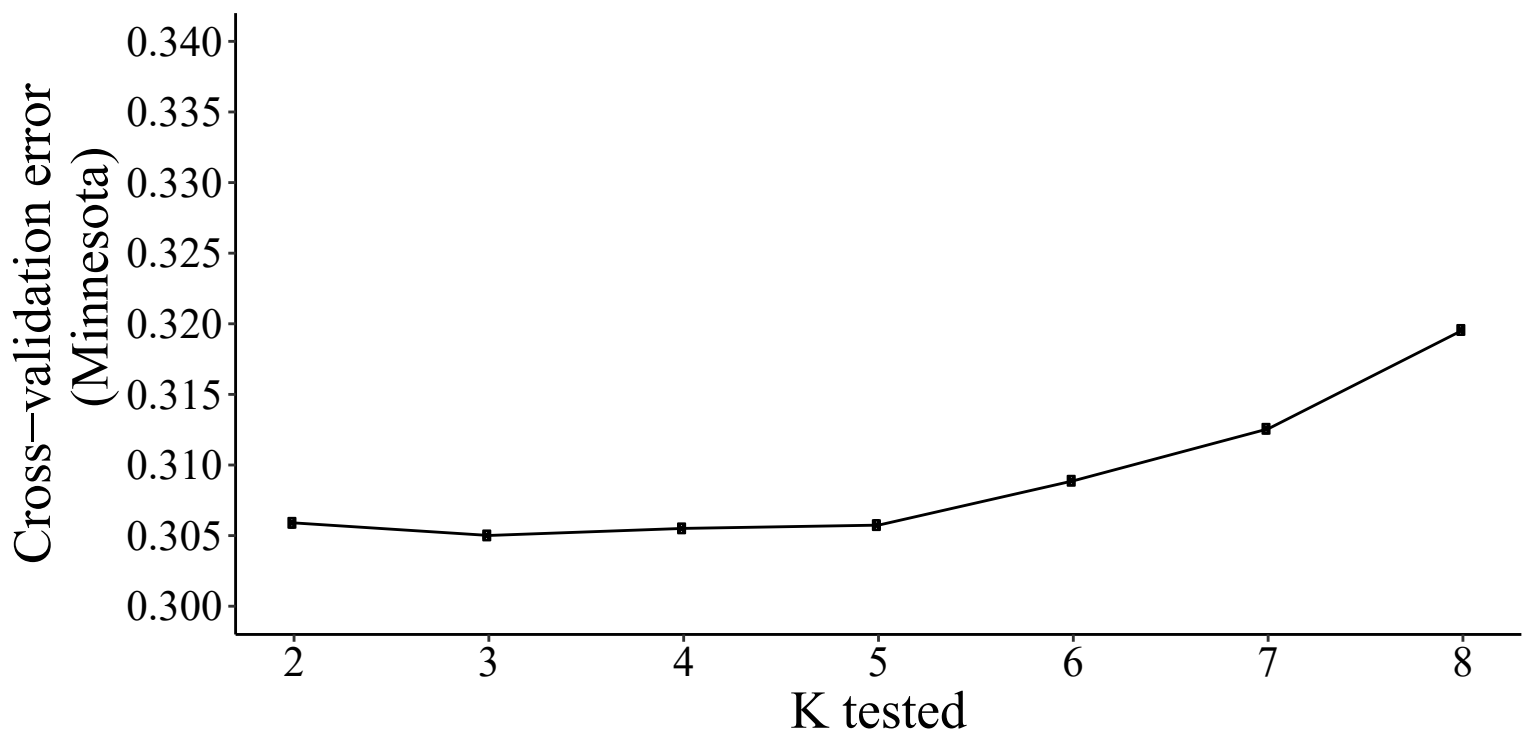

Supplement: Supplementary file 4 — Fig S3 [file EVA-14-1124-s005.pdf]

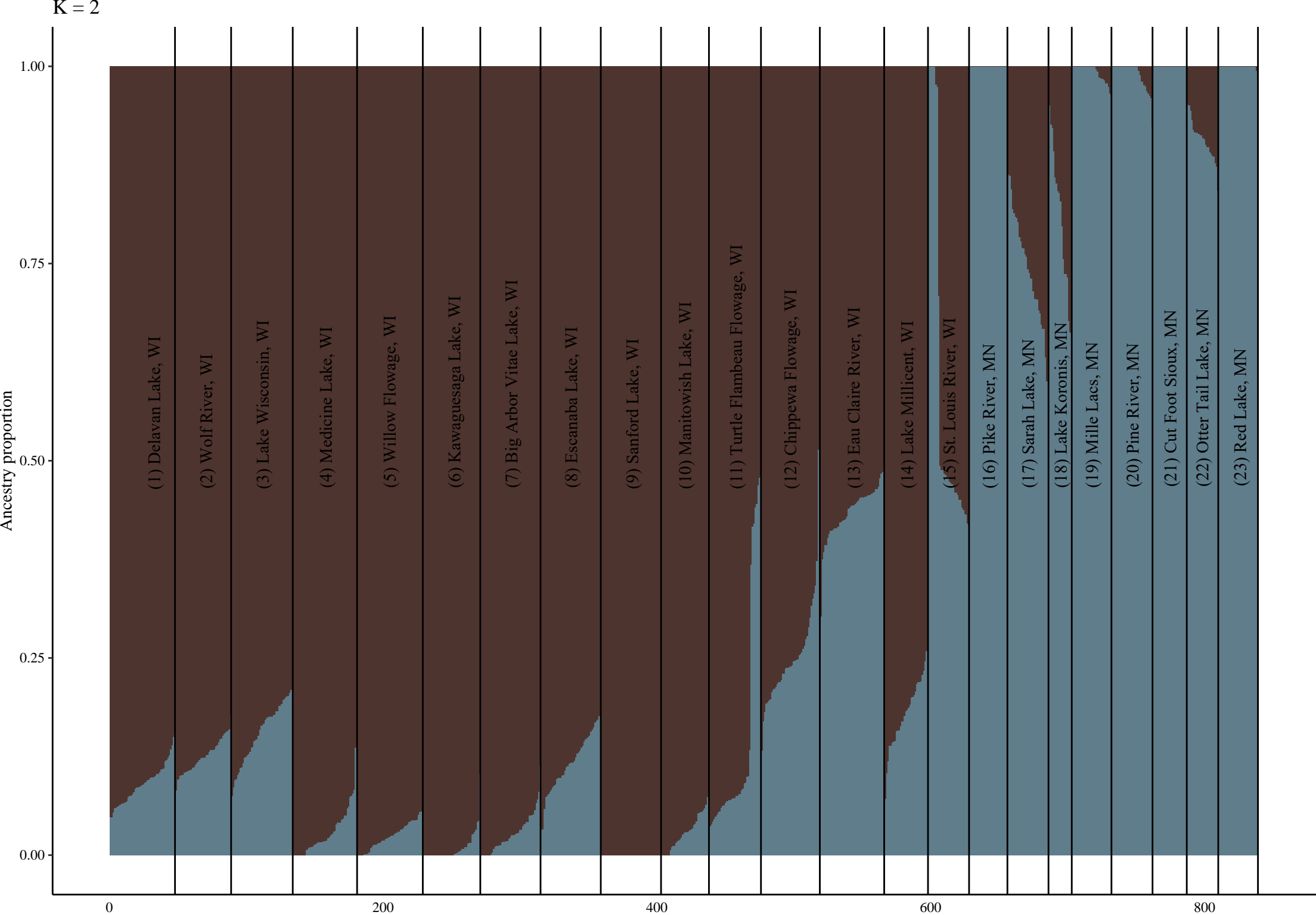

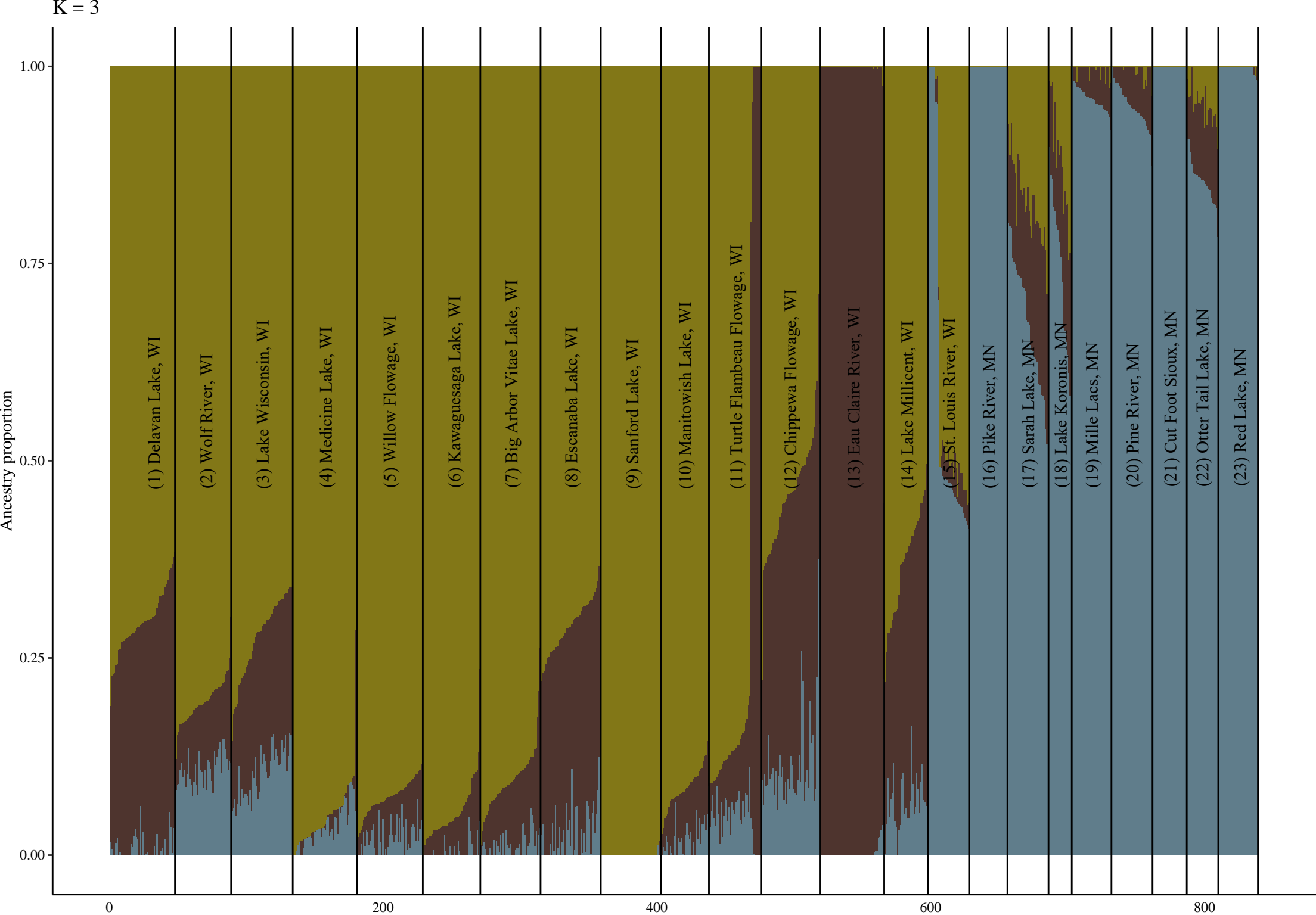

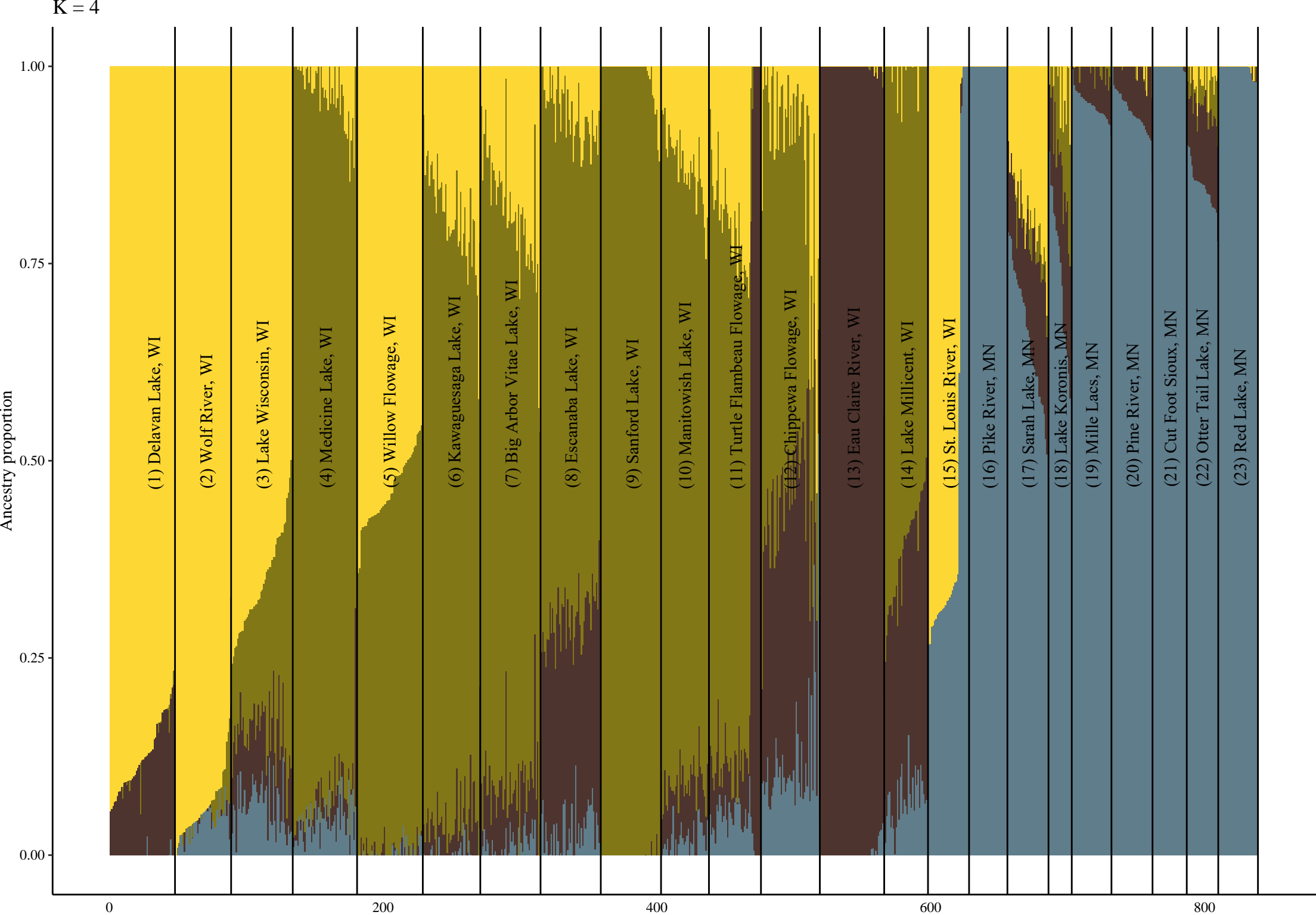

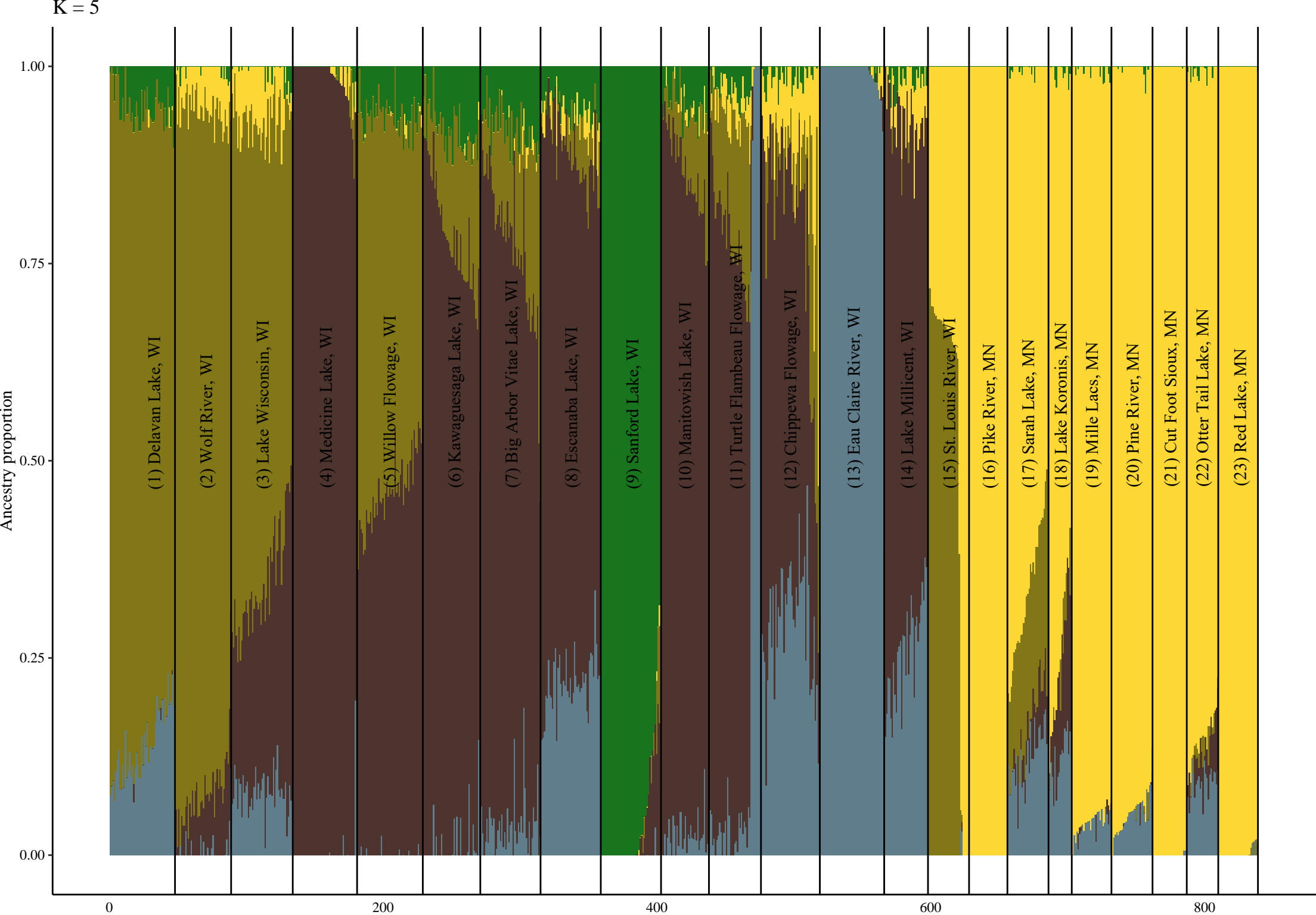

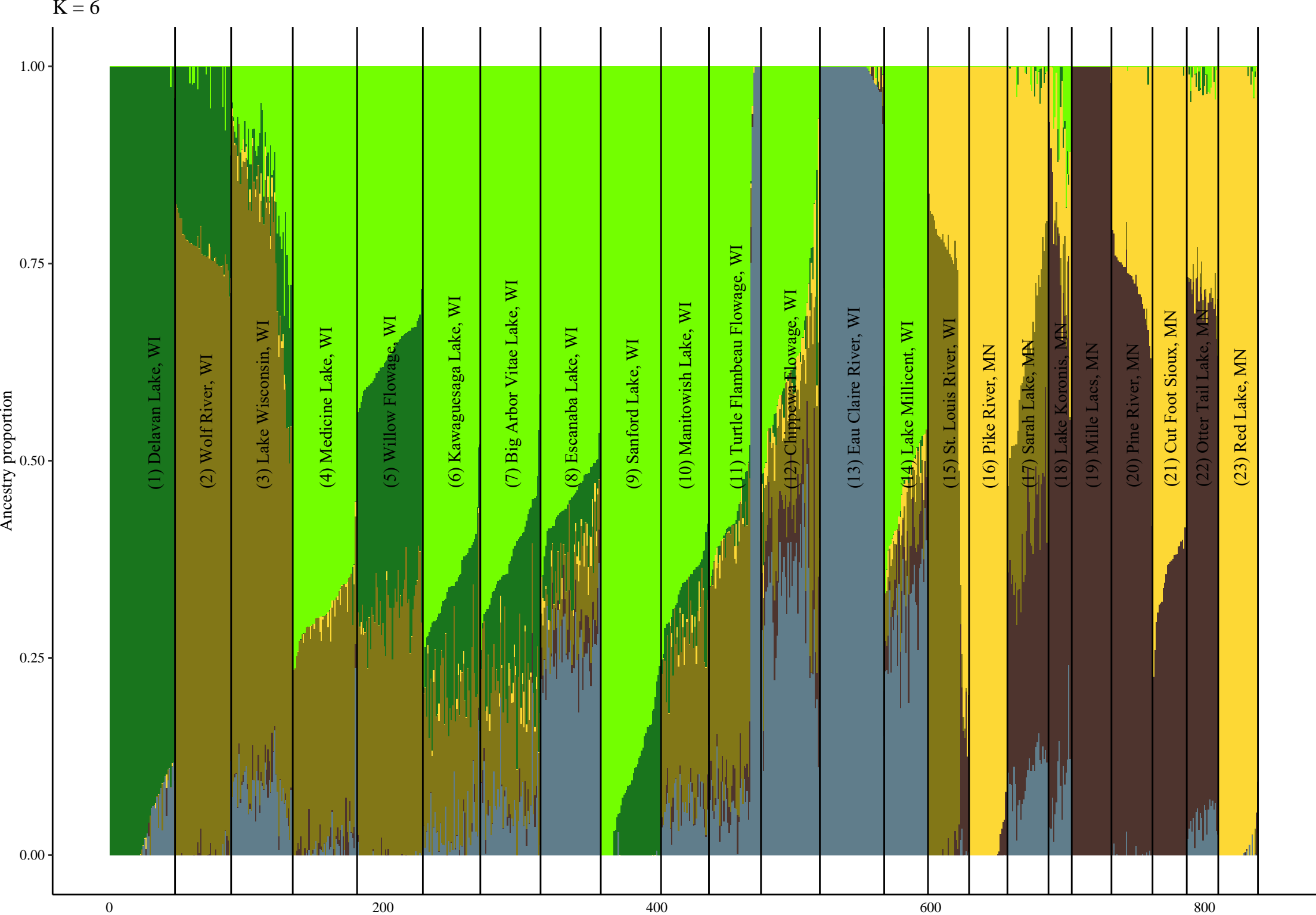

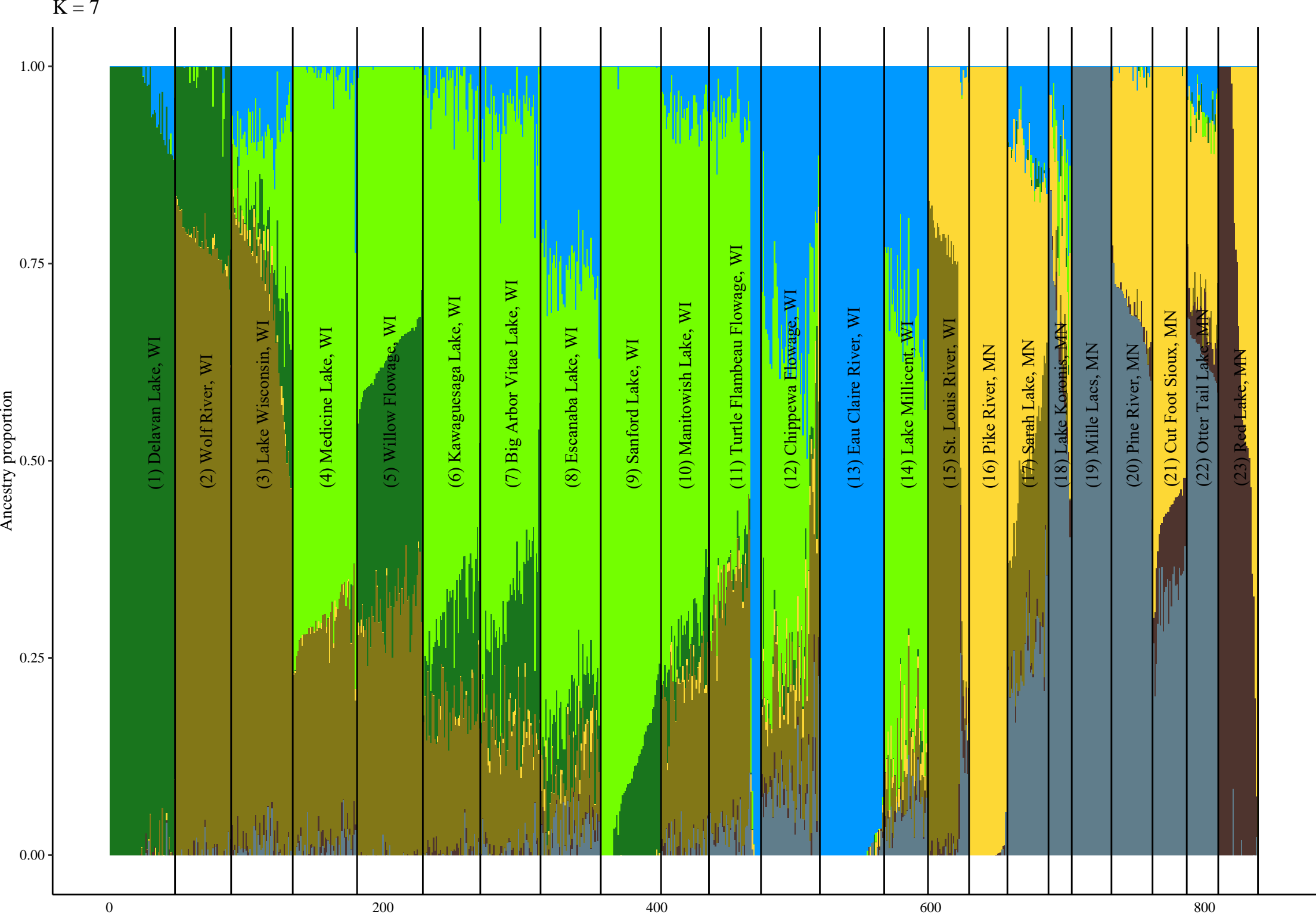

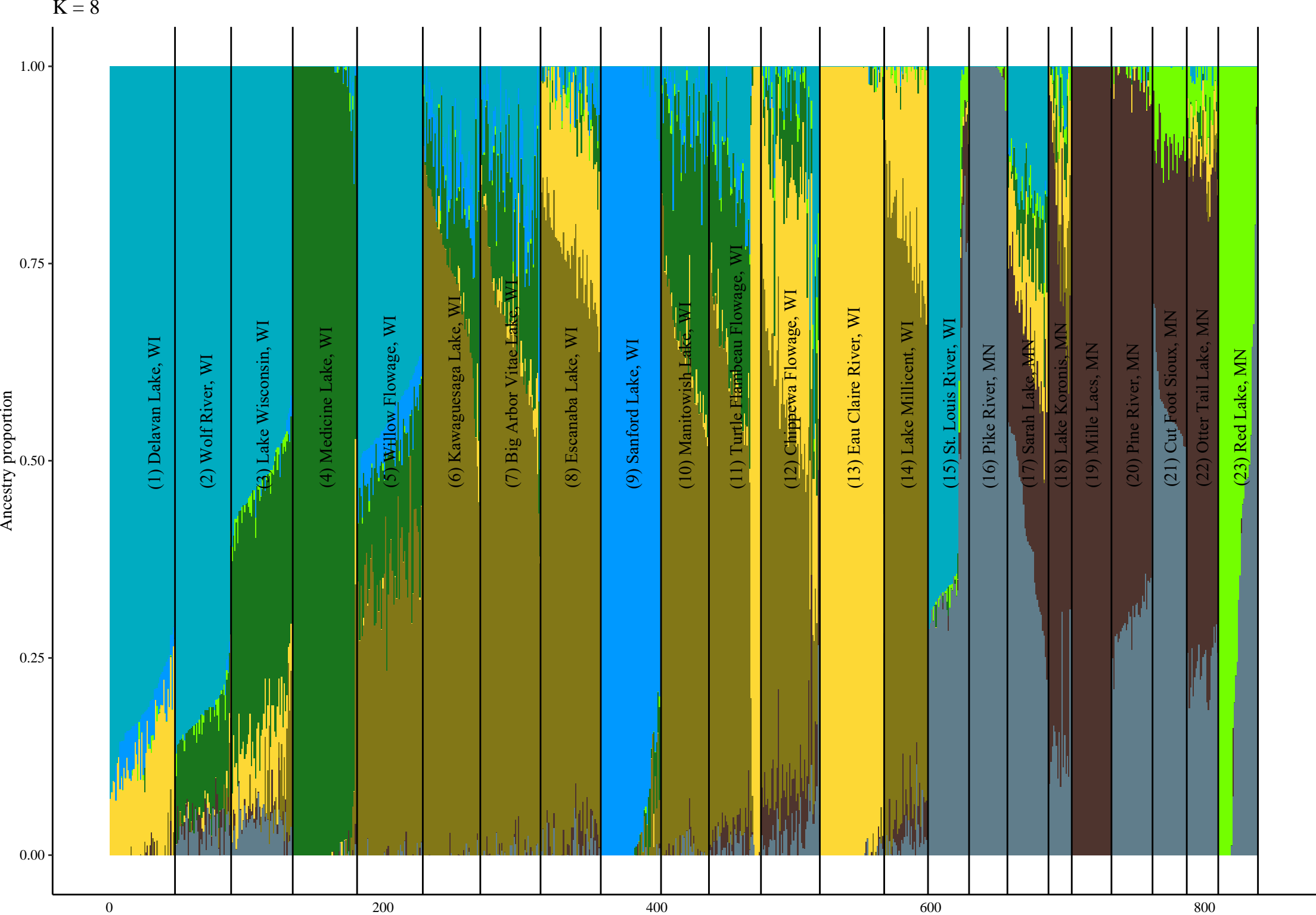

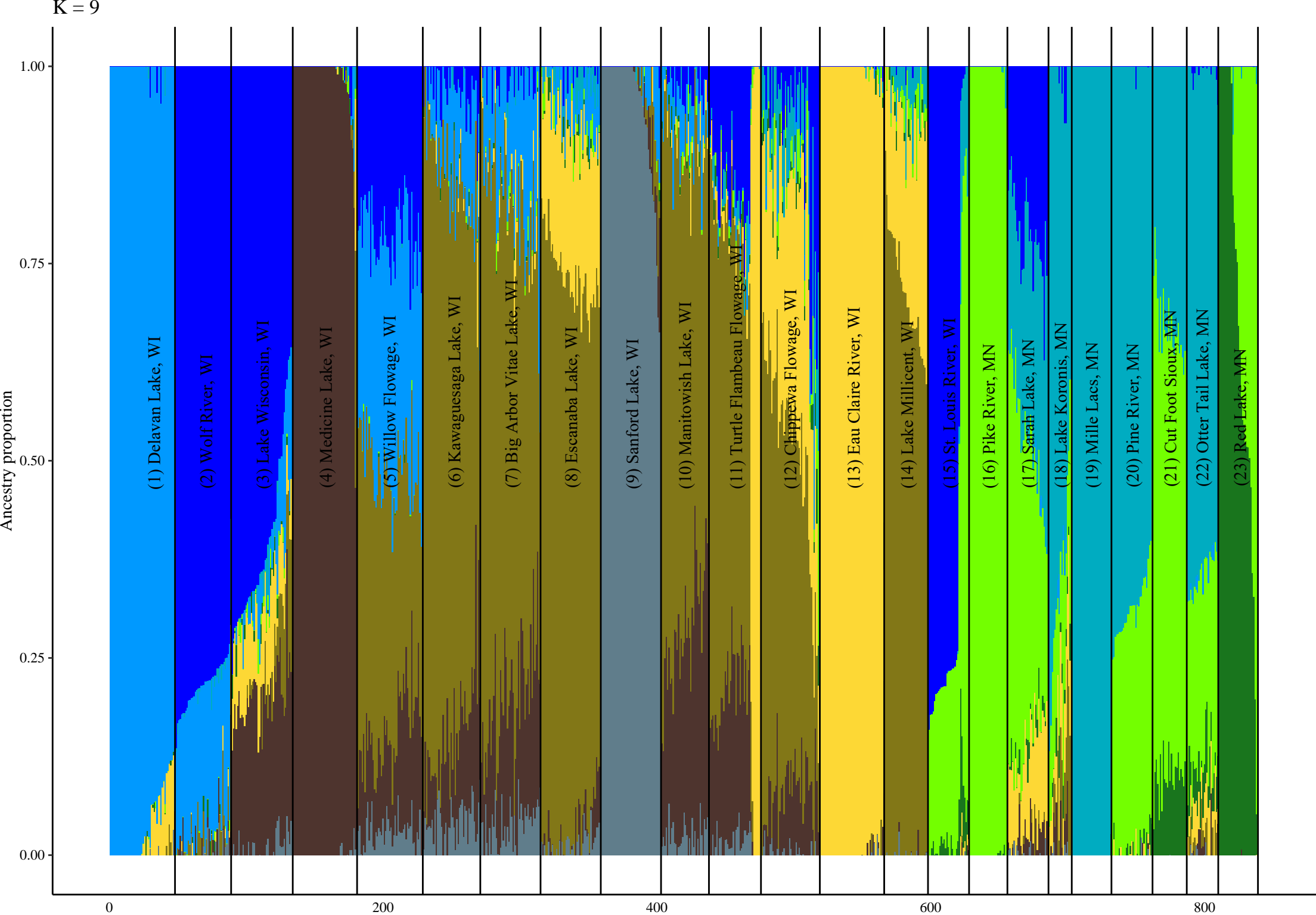

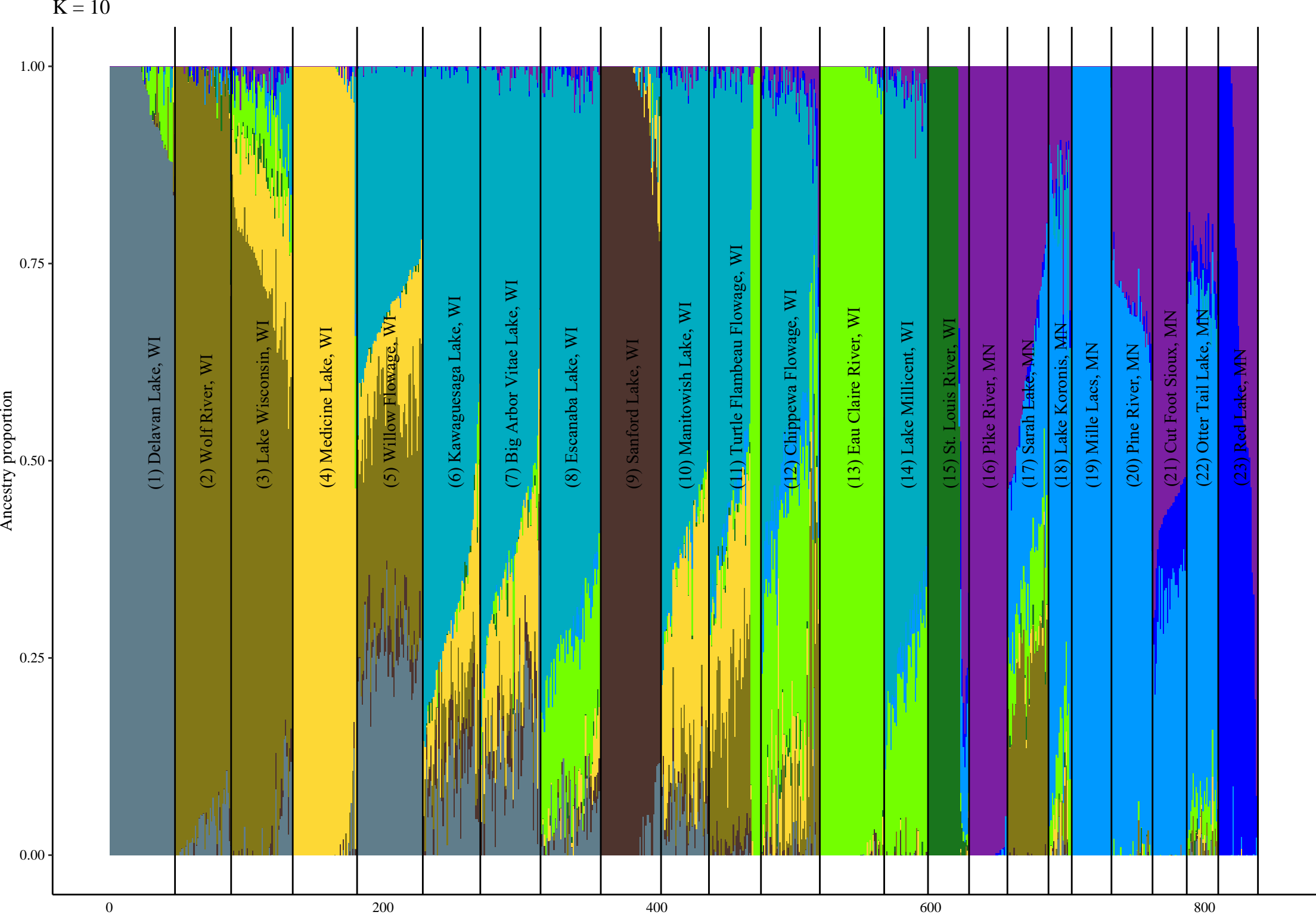

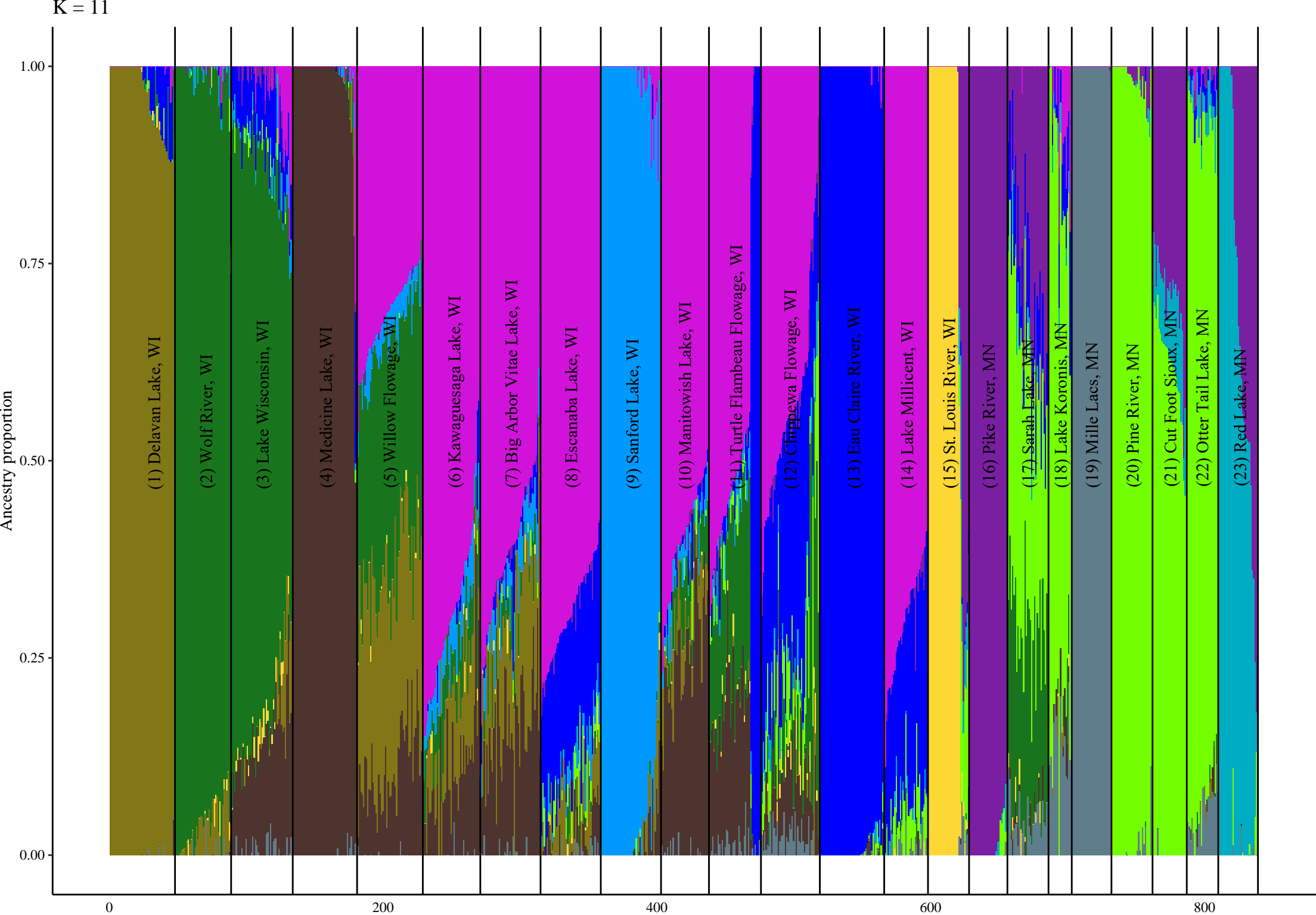

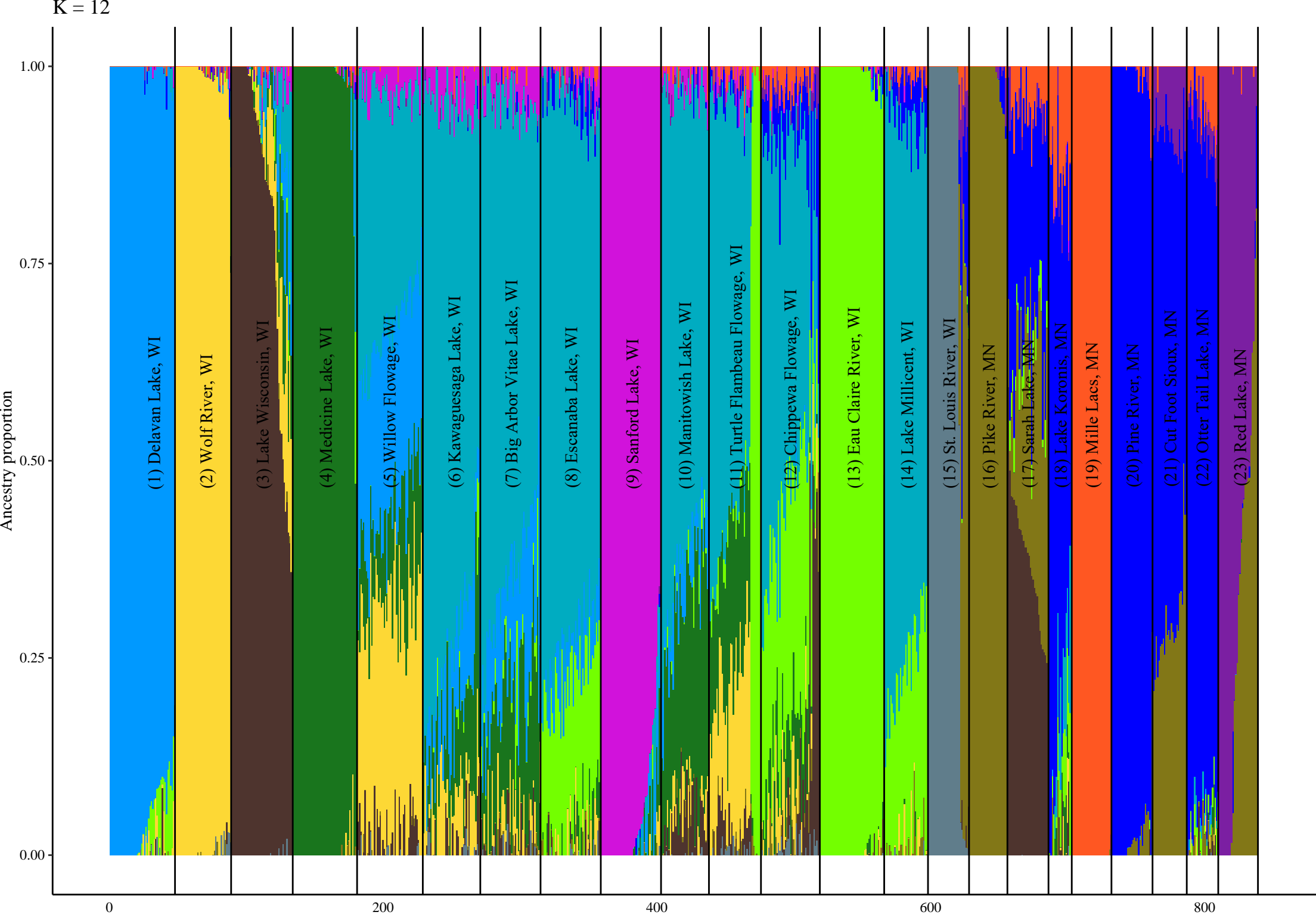

K = 13

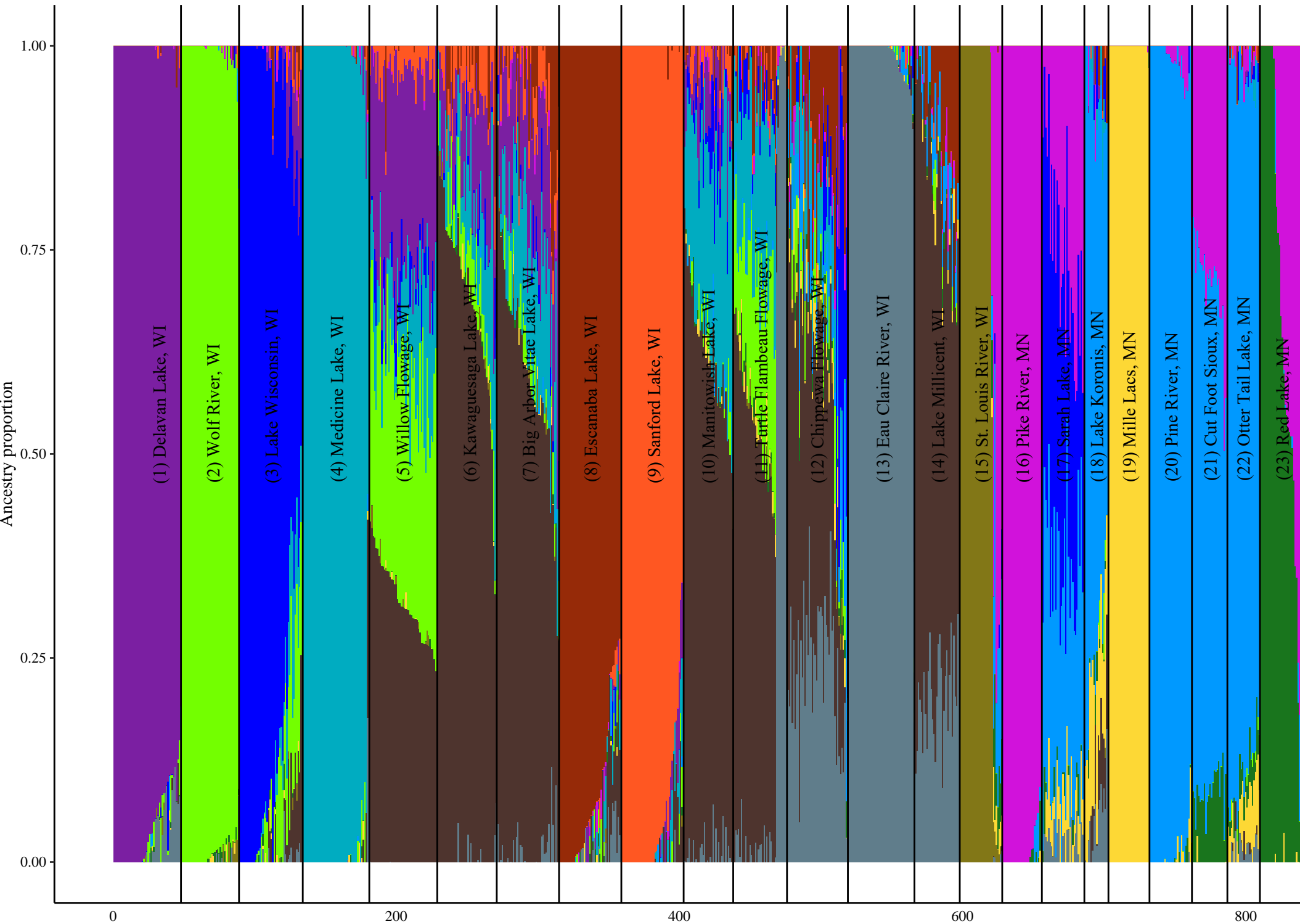

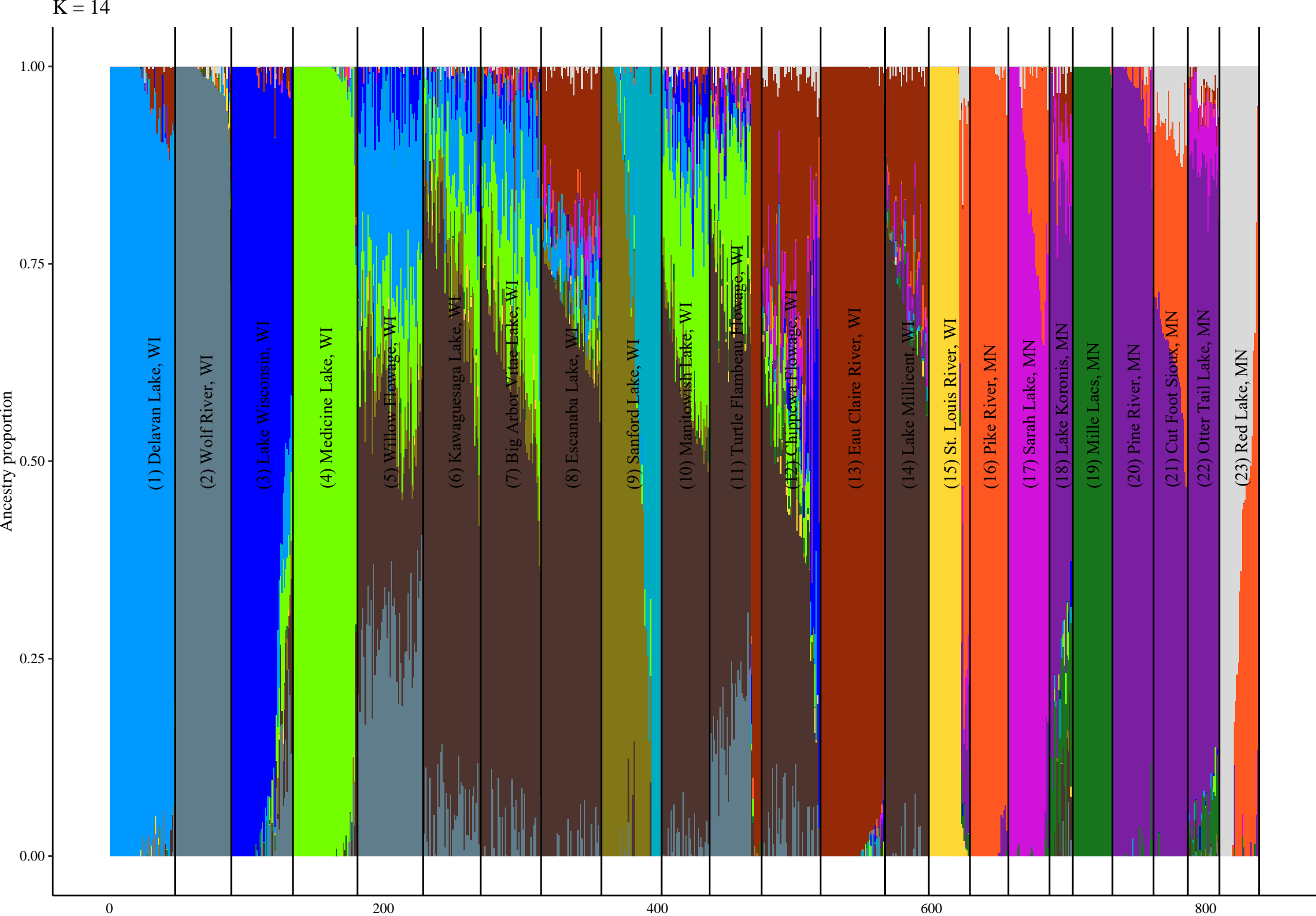

Supplement: Supplementary file 5 — Fig S4 [file EVA-14-1124-s002.pdf]
